# Human Artificial Chromosomes that Bypass Centromeric DNA

**DOI:** 10.1016/j.cell.2019.06.006

**Published:** 2019-07-25

**Authors:** Glennis A. Logsdon, Craig W. Gambogi, Mikhail A. Liskovykh, Evelyne J. Barrey, Vladimir Larionov, Karen H. Miga, Patrick Heun, Ben E. Black

**Affiliations:** 1 Department of Biochemistry and Biophysics, Graduate Program in Biochemistry and Molecular Biophysics, and Epigenetics Institute, Perelman School of Medicine, University of Pennsylvania, Philadelphia, PA 19104, USA; 2 Developmental Therapeutics Branch, National Cancer Institute, Bethesda, MD 20892, USA; 3 Wellcome Trust Centre for Cell Biology, Institute of Cell Biology, School of Biological Sciences, University of Edinburgh, Edinburgh EH9 3BF, UK; 4 Center for Biomolecular Science and Engineering, University of California, Santa Cruz, Santa Cruz, CA 95064, USA

**Keywords:** centromere, human artificial chromosome, HAC, kinetochore, nucleosome, histone, chromatin, epigenetics, mitosis, synthetic chromosome

## Abstract

Recent breakthroughs with synthetic budding yeast chromosomes expedite the creation of synthetic mammalian chromosomes and genomes. Mammals, unlike budding yeast, depend on the histone H3 variant, CENP-A, to epigenetically specify the location of the centromere—the locus essential for chromosome segregation. Prior human artificial chromosomes (HACs) required large arrays of centromeric α-satellite repeats harboring binding sites for the DNA sequence-specific binding protein, CENP-B. We report the development of a type of HAC that functions independently of these constraints. Formed by an initial CENP-A nucleosome seeding strategy, a construct lacking repetitive centromeric DNA formed several self-sufficient HACs that showed no uptake of genomic DNA. In contrast to traditional α-satellite HAC formation, the non-repetitive construct can form functional HACs without CENP-B or initial CENP-A nucleosome seeding, revealing distinct paths to centromere formation for different DNA sequence types. Our developments streamline the construction and characterization of HACs to facilitate mammalian synthetic genome efforts.

## Highlights

• Development of human artificial chromosomes (HACs) where CENP-A chromatin is seeded
• Seeding CENP-A nucleosome assembly induces centromere formation
• Seeding centromeric chromatin bypasses sequence elements in repetitive centromere DNA
• Non-repetitive HAC templates ease initial construction and downstream genomic analyses

## Graphical Abstract

**Figure:**
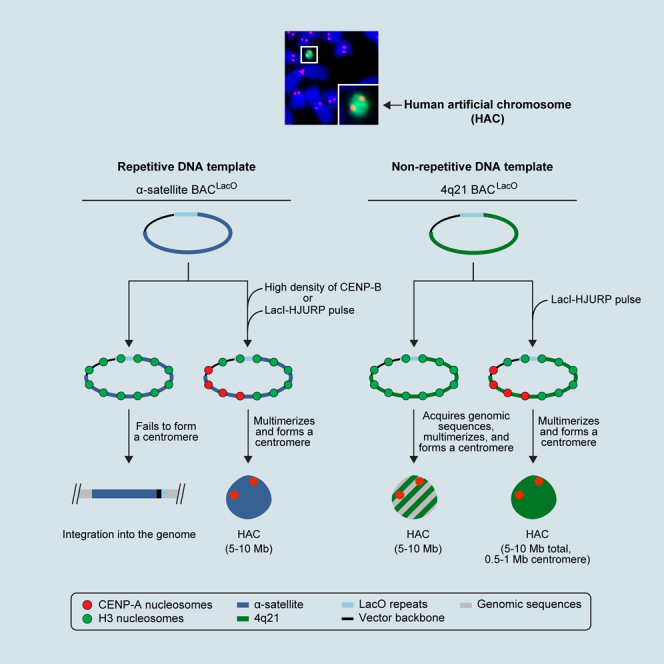

## Teaser

Development of human artificial chromosomes that bypass centromeric DNA removes a key barrier limiting mammalian synthetic genome efforts.

## Introduction

Artificial chromosomes, either those built from isolated (Schueler et al., 2001) or synthetic (Basu et al., 2005, Ohzeki et al., 2002, Richardson et al., 2017) sequences, have the potential to transform synthetic biology and permit the development of numerous radical advancements in medicine (Boeke et al., 2016). The early stages of an ambitious project to generate an entire set of synthetic human chromosomes, termed the Human Genome Project-Write (Boeke et al., 2016), is building on recent success with synthetic budding yeast chromosomes (Annaluru et al., 2014, Richardson et al., 2017). Among many potential hurdles to translate success from yeast to mammals, the centromere likely represents the biggest challenge. Centromeres are the loci present once per natural chromosome that guide their segregation at cell division (McKinley and Cheeseman, 2016). While in budding yeast these loci are small (∼125 bp) genetic elements, most other eukaryotes, including mammals, have an essential epigenetic contribution to their specification. This has provided an explanation to the originally paradoxical observation that the DNA typically found at human centromeres (α-satellite) is neither necessary nor sufficient for centromere identity and function (Eichler, 1999). For instance, centromere sequences can be silent (e.g., on one of the two megabase-sized regions of α-satellite on a so-called pseudodicentric chromosome) (Earnshaw and Migeon, 1985, Warburton et al., 1997) or completely bypassed when a new centromere (e.g., a neocentromere) (Depinet et al., 1997, Hasson et al., 2011, du Sart et al., 1997, Warburton et al., 1997) is formed. Instead, nucleosomes in which the histone variant, CENP-A, replaces canonical H3 epigenetically specify centromere location (Black and Cleveland, 2011, McKinley and Cheeseman, 2016).

Human artificial chromosomes (HACs) were first generated more than 20 years ago (Harrington et al., 1997), and through subsequent innovations, it became clear that the establishment of centromeric chromatin with CENP-A nucleosomes is what defines a functional HAC (Ebersole et al., 2000, Grimes et al., 2002, Ikeno et al., 1998, Mejía et al., 2002, Ohzeki et al., 2012, Okada et al., 2007). After the rare instance when a functional centromere is established, it is then faithfully propagated through the well-established epigenetic pathway that includes the dedicated centromere chromatin assembly protein, HJURP (Dunleavy et al., 2009, Foltz et al., 2009) (for a review, see McKinley and Cheeseman, 2016). A non-essential centromere protein, CENP-B—the only known sequence-specific DNA binding protein at mammalian centromeres, recognizing the 17-mer “CENP-B box” recognition element—plays an essential role in HAC formation (Ohzeki et al., 2002, Okada et al., 2007). This is presumably through its interactions with the CENP-A nucleosome and the key centromere protein, CENP-C (Fachinetti et al., 2013, Fachinetti et al., 2015). Indeed, a classic study using the repetitive centromere DNA from the X chromosome found that only regions of α-satellite with a high density of functional CENP-B boxes generated functional HACs (Schueler et al., 2001). These and other findings have led to two assumed universal rules for HAC formation: (1) a requirement for the specific forms of α-satellite with a high density of CENP-B boxes (Ohzeki et al., 2002, Schueler et al., 2001), and (2) the expression of CENP-B (Okada et al., 2007).

Bypassing these two rules (e.g., by forming a HAC on non-repetitive DNA constructs) would have several clear benefits. First, HAC construction would be greatly facilitated. Traditional HACs contain 50–200 kb of highly repetitive DNA (Ebersole et al., 2000, Grimes et al., 2002, Ikeno et al., 1998, Mejía et al., 2002, Ohzeki et al., 2012, Okada et al., 2007), which greatly complicates handling at all steps, from their initial construction to their clonal stability during bacterial propagation. Second, mapping the chromatin features of HACs using sequencing-based approaches would become possible. For instance, it is imperative to know where functional centromeres are located relative to other functional genetic elements that the HACs are engineered to carry. The highly repetitive sequences on traditional HACs unfortunately prohibit any useful genomic methodologies to define their composition and organization. Third, non-repetitive sequences would allow mammalian synthetic chromosomes to be generated by employing some of the fundamental principles used in recent yeast synthetic chromosome construction, where DNA repeats were removed to make their designed sequences compatible with recombination-based assembly (Richardson et al., 2017).

In considering a new generation of HAC design, alternative systems have emerged to form new centromeres through the artificial seeding of nascent CENP-A nucleosomes (Barnhart et al., 2011, Chen et al., 2014, Hori et al., 2013, Logsdon et al., 2015, Mendiburo et al., 2011, Ohzeki et al., 2012, Tachiwana et al., 2015) (reviewed in Barrey and Heun, 2017). One of these approaches, in fruit fly cells, built upon the earlier notion of epigenetic centromeric chromatin spreading (Maggert and Karpen, 2001). Initial CENP-A nucleosome assembly targeted locally at an array of Lac operator (LacO) sites eventually led to spreading of the centromere via natural centromeric chromatin assembly to the remainder of a small plasmid that did not contain any natural centromeric sequences (Mendiburo et al., 2011). While this plasmid does not align on the metaphase plate at cell division and does not yield very high stability through cell divisions (compared to HACs, for instance), it formed a functional mitotic kinetochore—the proteinaceous complex that forms at a mitotic centromere—and directed interactions with the microtubule-based spindle (Mendiburo et al., 2011). Taken together, these studies open the possibility that the requirements of α-satellite DNA and CENP-B for HAC formation, mentioned above, could be circumvented.

Here, we improve HAC technology with a collection of HACs that include repetitive centromeric sequences or non-repetitive genomic sequences, testing each type for their dependence on seeding CENP-A nucleosome assembly. We employ gene editing of centromere components to elucidate the molecular requirements for the establishment and propagation of different types of HAC DNA templates, and we utilize genomic approaches to gain a highly resolved understanding of HAC copy number as well as genetic and epigenetic composition.

## Results

### Seeding HACs with CENP-A Nucleosomes

**Figure 1:**
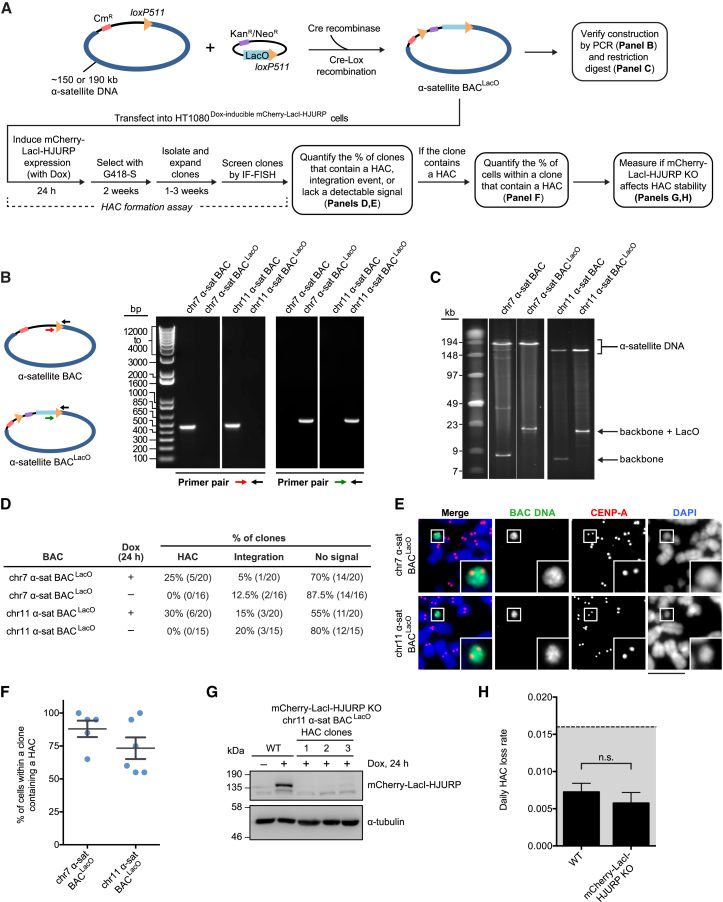
HAC Formation Is Stimulated by Seeding a Round of CENP-A Nucleosome Assembly with HJURP (A) Steps in building and testing HACs. (B) PCR analysis of α-satellite BAC^LacO^ constructs. (C) Restriction digest of BAC^LacO^ constructs to liberate individual parts. (D) Results of the HAC formation assays. (E) Representative images of chr7 and chr11 α-satellite BAC^LacO^ HACs. Insets are 2.5× magnifications. Bar, 10 μm. (F) Quantification of the percentage of cells containing an α-satellite BAC^LacO^ HAC within each HAC-positive clone. The mean value (± SEM) is shown for each BAC^LacO^ construct. (G) Immunoblots of the indicated cell lines. (H) Quantification of the daily HAC loss rate in WT or mCherry-LacI-HJURP KO cells after culturing without G418-S for 60 days. The mean daily loss rate (± SEM) is shown. n = 60 WT cells and 180 mCherry-LacI-HJURP KO cells, pooled from three independent experiments for each indicated cell type. The WT cells are from a chr11 α-satellite BAC^LacO^ clone, and the mCherry-LacI-HJURP KO cells are pooled from three derivative chr11 α-satellite BAC^LacO^ clones. n.s., not significant.

We first generated BAC constructs containing α-satellite sequences that are deemed nonfunctional in natural chromosomes due to a low density of CENP-B boxes (Hayden et al., 2013). A successful strategy to make these sequences functional to form a HAC is to first manipulate the constructs to increase the density of CENP-B boxes (Hayden et al., 2013). We devised an alternative strategy to avoid manipulation of the α-satellite sequences, themselves, by artificially driving an initial round of CENP-A chromatin assembly on an adjacent site on the construct. Our general strategy was to assemble constructs consisting of BACs harboring an array of LacO repeats immediately adjacent to human genomic DNA sequences (hereafter termed BAC^LacO^) (Figure 1A). Then, to the LacO array, we targeted mCherry-LacI-HJURP, inducibly expressed from a genomically integrated transgene. This targeting would potentially initiate the assembly of CENP-A nucleosomes directly onto the BAC and facilitate the spreading of CENP-A nucleosomes to the neighboring sequences (Mendiburo et al., 2011). We engineered two BAC^LacO^ vectors containing α-satellite DNA coming from CENP-A-poor regions of the centromere on chromosomes (chr) 7 and 11 (Figure 1A). Our cloning strategy positioned the LacO repeats within 300 bp of the α-satellite sequence, keeping this distance small to potentially permit efficient spreading of centromeric chromatin. We isolated α-satellite BAC^LacO^ constructs that successfully recombined (Figure 1B) and retained both the repetitive α-satellite and LacO arrays (Figure 1C). Using established methodologies to isolate and identify HACs (Grimes et al., 2002, Okamoto et al., 2007), we found that a pulse of mCherry-LacI-HJURP expression was sufficient to stimulate HAC formation (Figures 1D and 1E). Because we obtained nearly identical results on two independent α-satellite sequences (Figures 1D–1F), we conclude that our strategy would stimulate HAC formation on broad classes of α-satellite higher order repeats. As expected, there was no HAC formation in the absence of the round of CENP-A chromatin assembly directed by the pulse of mCherry-LacI-HJURP (Figure 1D), indicating that the presence of the LacO array, itself, does not drive centromere formation on BAC^LacO^ constructs.

The HACs we formed were highly penetrant within a clonal cell population (Figure 1F), likely due to the pulse of mCherry-LacI-HJURP driving efficient and rapid centromere acquisition that can then be propagated independently of the initial HJURP-mediated seeding of CENP-A nucleosome assembly. Alternatively, we considered that low, leaky expression of mCherry-LacI-HJURP continues to drive CENP-A nucleosome assembly on the HAC, thereby stabilizing the HAC in the cell. Therefore, we tested if genetically ablating mCherry-LacI-HJURP expression via CRISPR/Cas9-mediated gene editing affects HAC stability (Figure 1A). Choosing a cell line in which the chr11 α-satellite BAC^LacO^ HAC is present in ≥95% of cells, we derived three monoclonal cell lines in which mCherry-LacI-HJURP expression has been disrupted (Figure 1G). Using the standard approach for measuring HAC maintenance (Nakano et al., 2008, Ohzeki et al., 2012, Schueler et al., 2001), wherein all clones were cultured without antibiotic selection (G418-S) for 60 days, we found that the absence of mCherry-LacI-HJURP did not affect the daily HAC loss rate (Figure 1H). These daily HAC loss rates are similarly low as those reported for “conventional” HACs (Figure 1H, the range is shaded in gray (Ebersole et al., 2000, Ikeno et al., 1998)). Thus, the action of seeding CENP-A nucleosome assembly is limited to centromere establishment. After that, the centromere on the HAC is epigenetically maintained in the same manner as on natural chromosomes.

### CENP-B-Independent HAC Formation and Maintenance

**Figure 2:**
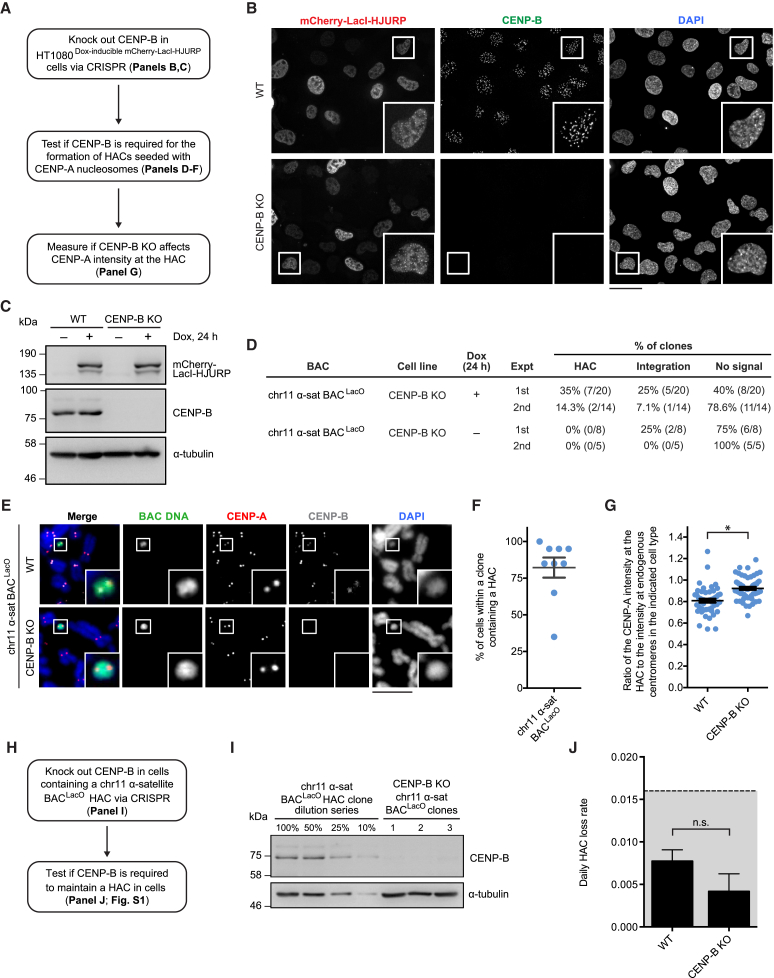
CENP-B Is Not Required for Formation or Maintenance of HACs Seeded with CENP-A Nucleosomes (A) Steps to test whether or not CENP-B participates in the formation of HACs seeded with CENP-A nucleosomes. (B) Representative images of the indicated cell lines following 24 h of dox treatment. Insets: 2.5× magnifications. Bar, 40 μm. (C) Immunoblots of the indicated cell lines. (D) Results of the HAC formation assays. (E) Representative images of HACs formed in WT and CENP-B KO cells. Insets: 2.5× magnifications. Bar, 10 μm. (F) Quantification of the percentage of CENP-B KO cells containing a HAC within each clone. The mean value (± SEM) is shown. (G) Quantification of CENP-A intensity at HACs formed in WT and CENP-B KO cells relative to the intensity at centromeres on endogenous chromosomes. The mean ratio (± SEM) is shown. n = 50 WT cells and 57 CENP-B KO cells, pooled from 3 independent clones for each indicated cell type. An asterisk indicates p < 0.05. (H) Steps to test if CENP-B is important for maintenance of HACs that formed upon seeding of CENP-A nucleosomes. (I) Immunoblots of the indicated cell lines. (J) Quantification of the daily HAC loss rate in WT or mCherry-Lac-HJURP KO cells after culturing without G418-S for 60 days (shading as in Figure 1H). The mean daily loss rate (± SEM) is shown. n = 60 WT cells and 180 CENP-B KO cells, pooled from 3 independent experiments for each indicated cell type. The WT cells are from a chr11 α-satellite BAC^LacO^ clone, and the CENP-B KO cells are pooled from three derivative chr11 α-satellite BAC^LacO^ clones. n.s., not significant. See also Figure S1.

We next directly tested whether CENP-B expression—one of the universal requirements for conventional HAC formation (Okada et al., 2007)—could be bypassed by seeding CENP-A nucleosome assembly. To do so, we disrupted the CENP-B gene prior to performing a new set of HAC formation assays (Figures 2A–2C). We found that chr11 α-satellite BAC^LacO^ HACs form in the absence of CENP-B (Figure 2D,E). Because HAC formation on this construct is dependent on induction of mCherry-LacI-HJURP (Figure 2D,E), we conclude that seeding CENP-A nucleosomes onto the α-satellite DNA bypasses the requirement of CENP-B for centromere formation. Further, the absence of CENP-B did not affect the high number of cells containing a HAC (Figure 2F) (82% ± 7% in CENP-B knockout [KO] cells versus 73% ± 8% in wild type [WT] cells, shown in Figure 1F) or substantially alter the amount of CENP-A on the centromere of the HAC relative to those on natural chromosomes (Figure 2G) (note there is a small but measurable increase in the CENP-B KO cells). Thus, our experiments indicate that the absence of CENP-B has no detectable negative effect upon forming a HAC via seeding of CENP-A nucleosomes.

**Figure S1:**
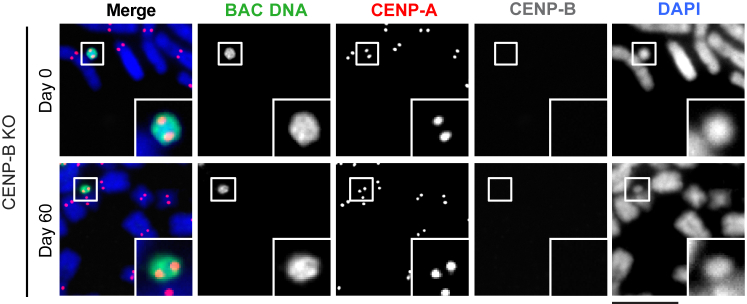
Chr11 α-Satellite BAC^LacO^ HACs Are Stably Propagated in CENP-B KO Cells, Related to Figure 2 Representative images of a chr11 α-satellite BAC^LacO^ HAC in CENP-B KO cells at the beginning of the HAC maintenance assay (Day 0) and after 60 days of culturing in the absence of G418-S (Day 60). Insets are 2.5× magnification. Bar, 10 μm.

Because prior efforts with conventional HACs failed to form any functional centromeres in the absence of CENP-B (Okada et al., 2007), there are no data to indicate whether or not CENP-B is also important for HAC maintenance. To address this issue, we performed a HAC maintenance assay with a cell line containing a chr11 α-satellite BAC^LacO^ HAC and three monoclonal cell line derivatives of it in which we disrupted the CENP-B gene (Figures 2H and 2I). We found that the absence of CENP-B did not affect the daily HAC loss rate of the α-satellite HACs (Figure 2J). Further, CENP-A was retained at the HAC in the absence of CENP-B through our 60-day assay (Figure S1). Thus, we conclude that CENP-B is also dispensable for the maintenance of a HAC.

### HACs that Lack α-Satellite DNA

**Figure S2:**
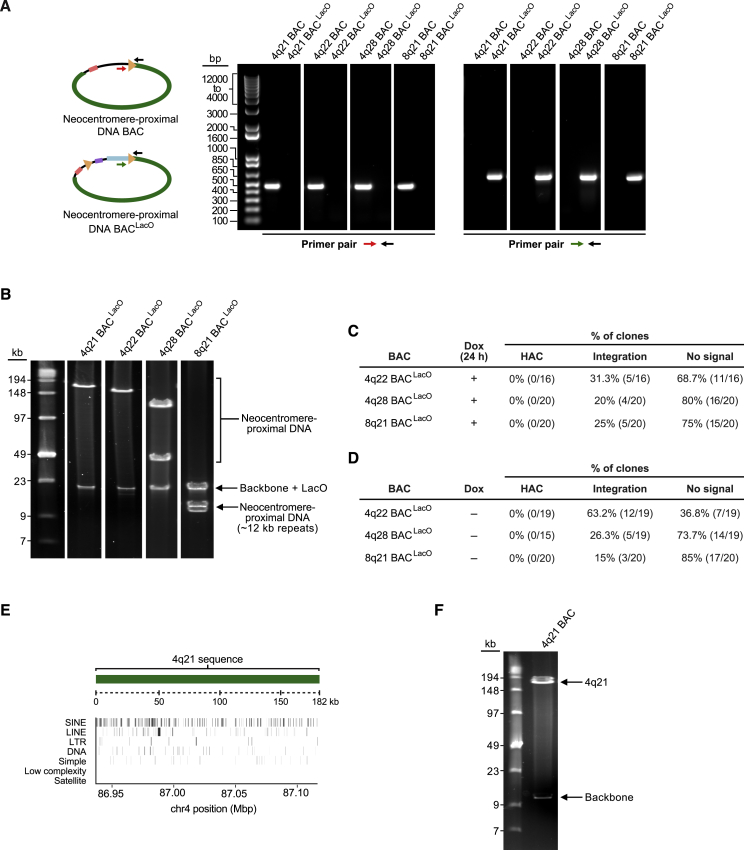
Non-α-satellite BAC Constructs Tested for HAC Formation, Related to Figure 3 (A) PCR analysis to confirm construction of the non-α-satellite BAC^LacO^ constructs. The red and black primer pair amplifies a 458 bp fragment only in the parental non-α-satellite BAC vector, and the green and black primer pair amplifies a 558 bp fragment only in the non-α-satellite BAC^LacO^ vector. (B) Restriction digest analysis with NotI showing 150-190 kb of non-α-satellite DNA is maintained in the non-α-satellite BAC^LacO^ vectors. C,D) Results of a HAC formation assays with a subset of non-α-satellite BAC^LacO^ constructs in doxycycline-inducible mCherry-LacI-HJURP HT1080 cells with a 24 h pulse of mCherry-LacI-HJURP expression (C) or without mCherry-LacI-HJURP expression (D). (E) Illustration of the repeat abundance and position along the 4q21 sequence. The repeat elements are dispersed along the 4q21 sequence and do not appear to cluster in regions enriched with CENP-A in the 4q21 HAC clones (Figures 5A and 5B), indicating that there is no strong correlation between repeat element and CENP-A location. (F) Restriction digest analysis with NotI on the 4q21 BAC construct showing that the 4q21 sequence and vector backbone are present in the BAC, while the LacO repeats have been removed.

**Figure 3:**
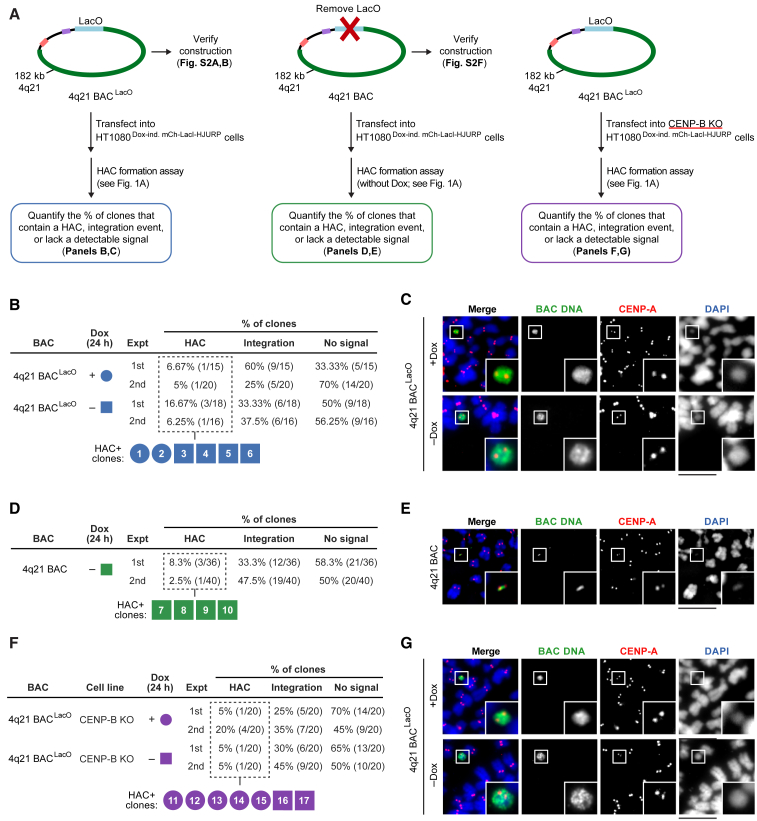
Formation of a HAC on a Template Lacking α-Satellite DNA Even without Seeding CENP-A Nucleosome Assembly or CENP-B (A) Three tests of a non-α-satellite sequence for its ability to form a HAC. (B) Results of the HAC formation assays with 4q21 BAC^LacO^ with and without seeding CENP-A nucleosome assembly. (C) Representative images of the 4q21 BAC^LacO^ HACs formed with and without seeding CENP-A nucleosome assembly. (D) Results of the HAC formation assays with the 4q21 BAC (i.e., a construct lacking any LacO repeats). (E) Representative images of the 4q21 BAC HACs formed without any residual CENP-A nucleosome seeding by mCherry-LacI-HJURP. (F) Results of the HAC formation assays with 4q21 BAC^LacO^ in CENP-B KO mCherry-LacI-HJURP HT1080 cells. (G) Representative images of the 4q21 BAC^LacO^ HACs formed in the CENP-B KO cells. Insets: 2.5× magnifications. Bar, 10 μm (C, E, and G). See also Figure S2 and Table S1.

The most prominent proposal for the role of α-satellite DNA in HAC formation is that a high density of CENP-B boxes facilitates early steps in centromere formation (Fujita et al., 2015, Ohzeki et al., 2002, Okada et al., 2007, Schueler et al., 2001). Because seeding CENP-A nucleosome assembly bypasses the requirement of CENP-B for centromere formation (Figure 2), we hypothesized that, likewise, the requirement for α-satellite DNA might be bypassed. To test this, we built and performed a small-scale HAC formation screen with a set of BACs containing an array of LacO repeats adjacent to non-α-satellite human genomic sequences (Figures S2A and S2B). We chose sequences for our initial screening based on proximity to known neocentromeres (Amor et al., 2004, Hasson et al., 2011, Hasson et al., 2013), and we also included a clone several Mbp distal to a well-studied neocentromere (PD-NC4) (Table S1). One construct in the screen, 4q21 BAC^LacO^, formed several HACs (Figures 3A–3C and S2C–S2E). In stark contrast to the α-satellite versions that we tested (Figures 1 and 2), we found that 4q21 BAC^LacO^ also reproducibly formed HACs in the absence of the induction of mCherry-LacI-HJURP expression (Figures 3A–3C). We considered that non-α-satellite sequences might be particularly sensitive to leaky expression of mCherry-LacI-HJURP in the absence of doxycycline. Thus, we generated a version of the 4q21 BAC that is identical to 4q21 BAC^LacO^ but lacks the LacO array (Figure S2F) and found that it also forms HACs (Figures 3A, 3D, and 3E). This eliminated the possibility of a dependence on any leaky mCherry-LacI-HJURP expression or on any other property imparted by the LacO array itself. Because the only sequences to-date to form a HAC in the absence of seeding CENP-A nucleosomes require CENP-B (Okada et al., 2007), we also considered the possibility that 4q21 BAC^LacO^ HACs somehow form via a CENP-B-dependent centromere formation pathway. To directly test this, we performed HAC formation assays with 4q21 BAC^LacO^ in our cell line where the CENP-B gene had been disrupted (Figures 2B and 2C) and found that HAC formation occurred in the absence of CENP-B (Figures 3A, 3F, and 3G). Thus, we conclude that the non-repetitive, non-centromeric 4q21 BAC^LacO^ construct forms a HAC in a CENP-B-independent manner. Taken together, this series of HAC formation assays with non-α-satellite DNA constructs clearly indicate that centromere formation must be different from the CENP-B-dependent pathway used by traditional HACs (Ebersole et al., 2000, Grimes et al., 2002, Harrington et al., 1997, Ikeno et al., 1998, Mejía et al., 2002, Ohzeki et al., 2002, Ohzeki et al., 2012, Okada et al., 2007, Schueler et al., 2001) or our new CENP-B-independent HACs that require seeding CENP-A nucleosome assembly (Figures 1 and 2).

### Multiple Pathways for HAC Formation on a Non-repetitive DNA Template

**Figure 4:**
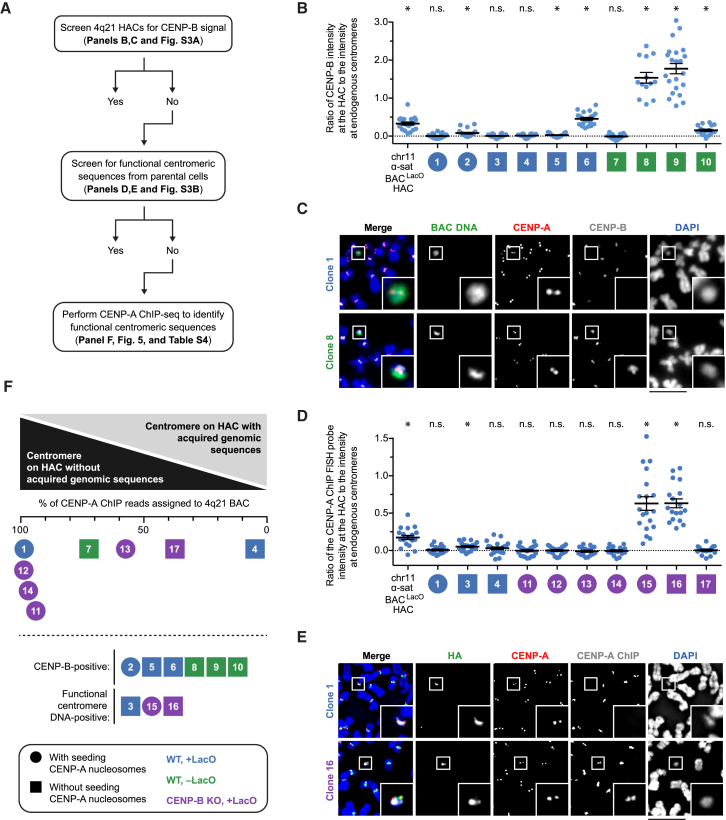
Seeding CENP-A Nucleosome Assembly Dictates the Pathway to Centromere Formation (A) Steps to test whether the 4q21 HACs have acquired CENP-B protein or functional α-satellite DNA. (B) Quantification of the intensity of CENP-B at chr11 α-satellite BAC^LacO^ and 4q21 HACs relative to the intensity at endogenous centromeres. Each data point represents a measurement taken at a single HAC. The mean ratio (± SEM) is shown. n = 20, 19, 20, 20, 20, 19, 21, 18, 13, 22, and 18 HACs for the clones shown, in order. p is < 0.0001, 0.8566, 0.0019, 0.6401, 0.2215, 0.0343, < 0.0001, 0.6269, < 0.0001, < 0.0001, < 0.0001 for the clones shown, in order, based on a one-sample t test with a hypothetical mean of 0. Clones with a p value < 0.05 are marked with an asterisk; clones with a p value ≥ 0.05 are marked as not significant (n.s.). (C) Representative images of a 4q21 HAC that has acquired CENP-B-bound sequences (clone 8) and one that has not (clone 1). (D) Quantification of the intensity of a CENP-A ChIP probe at chr11 α-satellite BAC^LacO^ and 4q21 HACs relative to the intensity at endogenous centromeres. Each data point represents a measurement taken at a single HAC. The mean ratio (± SEM) is shown. n = 20, 18, 20, 22, 22, 20, 18, 19, 19, 18, and 19 HACs for the clones shown, in order. p is < 0.0001, 0.5642, 0.0005, 0.1028, 0.9098, 0.9602, 0.4708, 0.7553, < 0.0001, < 0.0001, 0.7278 for the clones shown, in order, based on a one-sample t test with a hypothetical mean of 0. Clones with a p value 0.05 are marked with an asterisk; clones with a p value ≥ 0.05 are marked as not significant (n.s.). (E) Representative images of a 4q21 HAC that has acquired CENP-A-associated sequences (clone 16) and one that has not (clone 1). The HACs are detected with HA-LacI, which binds the LacO repeats present in the HACs. Insets: 2.5× magnifications. Bar, 10 μm (C and E). (F) Summary of the quantitative analysis of all 4q21 HAC clones. See also Figure S3 and Table S2.

We developed a tripartite strategy (Figure 4A) to investigate the pathway for centromere formation for each of the 17 clones isolated through our collection of 4q21-based HAC experiments (Figure 3) (clones 1–6 from 4q21 BAC^LacO^ in WT cells; clones 7–10 from 4q21 BAC in WT cells; clones 11–17 from 4q21 BAC^LacO^ in CENP-B KO cells).

**Figure S3:**
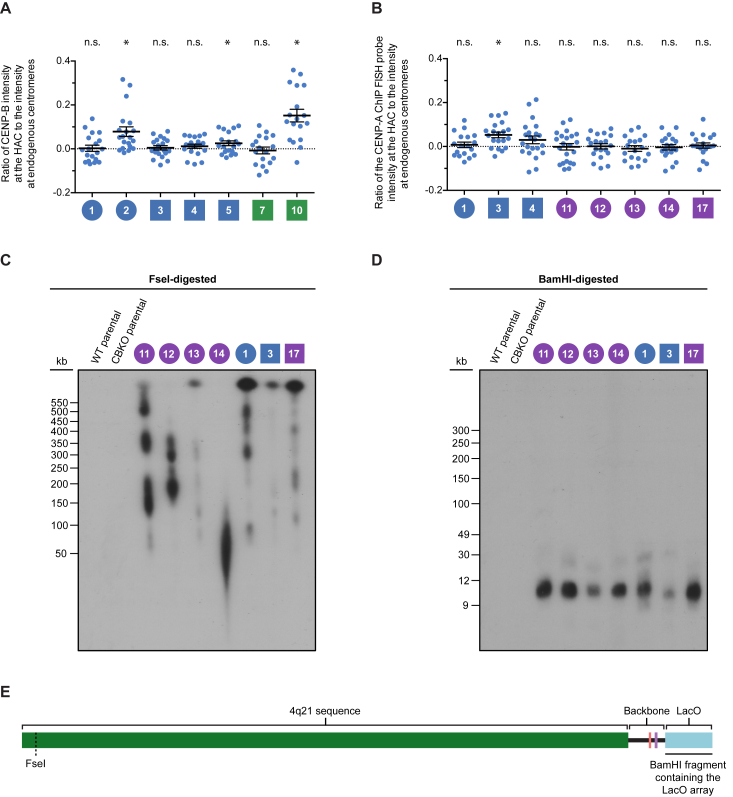
Analysis of the Centromeric Protein and Sequence Abundance, as well as the Organization, of Various 4q21 BAC^LacO^ HAC Clones, Related to Figure 4 A,B) Plot with an expanded y axis of the ratio of CENP-B (A) and CENP-A ChIP FISH probe (B) intensity at the HAC relative to endogenous centromeres for clones with a mean below 0.2; related to Figures 4B and 4D. Clones with a p value < 0.05 are marked with an asterisk; clones with a p value ≥ 0.05 are marked as not significant (n.s.). C,D) Southern blot analysis of the indicated cell lines showing variable sequence organization within the 4q21 HACs. Genomic DNA from each cell line was digested with the indicated restriction enzyme, separated by pulsed-field gel electrophoresis, transferred to a membrane, and hybridized with a LacO-specific probe. The FseI restriction enzyme digests the 4q21 BAC^LacO^ sequence one time; therefore, if the HAC had undergone a simple amplification of the 4q21 BAC^LacO^ sequence, multiples of a 203 kb band should be observed. However, we observed varying band sizes (C), indicating that each HAC had undergone structural rearrangements during HAC formation, which has been previously observed with α-satellite HACs (Kouprina et al., 2012). In all HACs assessed, the LacO array was largely intact (D), indicating that the rearrangements occurred in the 4q21 and backbone sequences within each HAC and not within the LacO array. (E) Restriction enzyme map of the FseI cut site and the fragment produced by BamHI enzyme digestion of the 4q21 BAC^LacO^ construct. BamHI cuts 26 other times throughout the 4q21 sequence and backbone (not shown), but these fragments are largely not detected by the LacO-specific probe (as shown in Panel D).

First, using immunofluorescence to detect CENP-B protein and fluorescence *in situ* hybridization (FISH) to detect the HACs, we found that four of the ten clones that formed in the WT (CENP-B-positive) background had no detectable CENP-B protein (Figures 4B, 4C, and S3A) (clones 1, 3, 4, and 7). The other six of the ten clones had detectable CENP-B, with widely varying levels of acquired native centromere sequences likely housing some or all of the functional centromeric chromatin.

Second, using FISH to detect functional centromeric chromatin on HACs detected with the expression of HA epitope-tagged LacI, we found that seven of the ten remaining clones generated with 4q21 BAC^LacO^ had no detectable acquisition of functional centromeric chromatin (Figures 4D, 4E, and S3B) (clones 1, 4, 11–14, and 17; note that clone 7 was generated with a 4q21 BAC construct that lacks a binding site for the HA epitope-tagged LacI, so it could not be included in the second step of our analysis). Two other HACs appeared to form with the acquisition of high levels of functional centromeric chromatin (clones 15 and 16) and another HAC formed with the acquisition of only very little functional centromeric chromatin (clone 3) (Figures 4D, 4E, and S3B).

Third, eight out of the original seventeen 4q21-based HACs whose formation could not be attributed to the acquisition of functional centromeric chromatin in the first two steps of our analysis were subjected to CENP-A chromatin immunoprecipitation sequencing (ChIP-seq) (Figure 4F). By comparing the reads in each HAC-containing cell line to the parental cell line lacking a HAC, we assigned all of the reads coming from the HAC to either the 4q21-containing BAC sequences or the rest of the human reference genome (Table S2). As with prior analysis of human neocentromeres (Hasson et al., 2013), there is a massive increase in CENP-A ChIP-seq reads from the functional centromere on the HAC relative to what is observed in parental cells lacking a HAC. Thus, we assigned all 4q21 CENP-A ChIP reads to the HAC. Using this strategy, we found that four of the HACs (clones 1, 11, 12, and 14) have centromeres residing on DNA essentially entirely comprised of 4q21-containing BAC sequences, while the other four (clones 4, 7, 13, and 17) have acquired genomic sequences upon which at least a portion of the functional centromere (defined by the presence of CENP-A nucleosomes) resides (Figure 4F; Table S2). Both types of HACs (those with centromeres exclusively on the 4q21 sequence and those with acquired genomic sequences) multimerized with rearrangements at unique locations relative to one another but always within non-repetitive regions (i.e., outside of the LacO array) of 4q21 BAC^LacO^ (Figures S3C–S3E).

**Figure 5:**
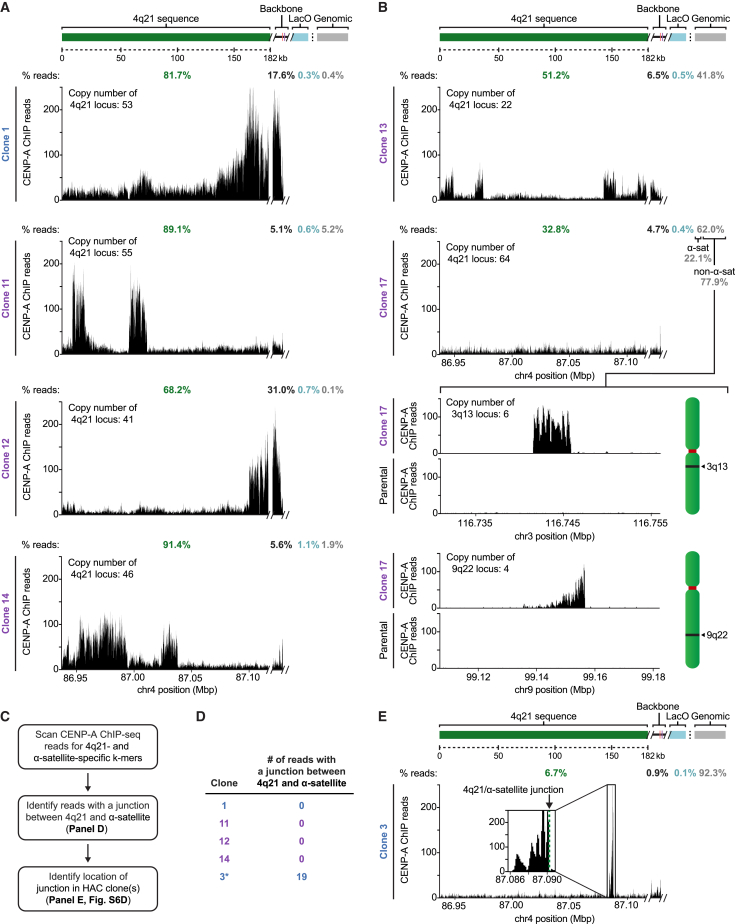
Genomic Analysis of 4q21 HACs Reveals Precise Location of Newly Formed Centromeres (A) CENP-A ChIP-seq analysis of the 4q21 HACs that formed without acquisition of CENP-A, functional α-satellite, or additional sequences from host chromosomes. CENP-A is localized along the 4q21 sequence and can be also be found on the backbone of the BAC. In all four clones, >90% of reads enriched with CENP-A align to the BAC sequence. The copy number of the 4q21 locus is shown for each HAC and includes the endogenous locus (that contributes 2.7 copies on average, as determined by IF-FISH). (B) CENP-A ChIP-seq analysis of two 4q21 HACs that had acquired additional sequences from the host chromosomes during HAC formation. In clone 13, the HAC had acquired sequences predominantly from the chr10 centromere, while clone 17 had acquired sequences from the chr10 centromere and two non-centromeric loci, 3q13 and 9q22. The copy number of the 4q21, 3q13, and 9q22 loci are shown for each HAC and includes the endogenous locus (that contributes 2.7, 4.0, and 3.2 copies on average for the 4q21, 3q13, and 9q22 loci, respectively, as determined by IF-FISH). (C) Steps to identify CENP-A ChIP-seq reads harboring a junction between 4q21 and α-satellite sequences in 4q21 HAC clones. (D) Summary of the read junction analysis showing that none of the 4q21 HAC clones that had formed without acquisition of CENP-A, functional α-satellite, or additional sequences from host chromosomes contained reads with a junction between 4q21 and α-satellite. However, a clone that had acquired functional centromeric chromatin (denoted by an asterisk) contained 19 reads with such junctions. (E) CENP-A ChIP-seq analysis of clone 3, which contains 19 reads spanning the junction between 4q21 and α-satellite (shown in Figure S6D). The location of the junction is indicated by a dashed green line. See also Figures S4 and S5 and Tables S3, S4, and S5.

**Figure S4:**
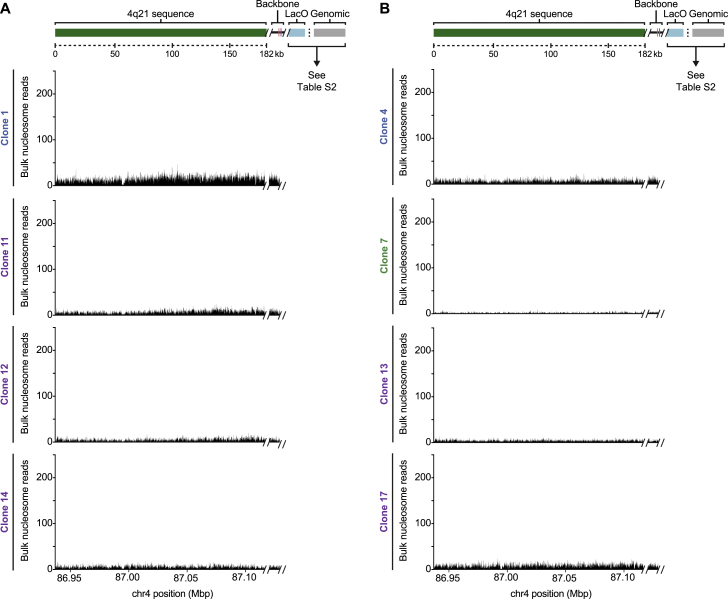
Analysis of Nucleosome-Associated Sequences in the 4q21 HACs, Related to Figure 5 A,B) Sequencing analysis of the bulk nucleosomal sequences associated with the 4q21 HACs, highlighting those that have not acquired sequences from host chromosomes (A) and those that have (B). The even distribution of reads along the 4q21 region indicates that this region consists of unique, complex sequences without amplification of specific regions. The clone-to-clone variation in read density across this region is due to amplification of the 4q21 sequence during HAC formation (Figure S5).

**Figure S5:**
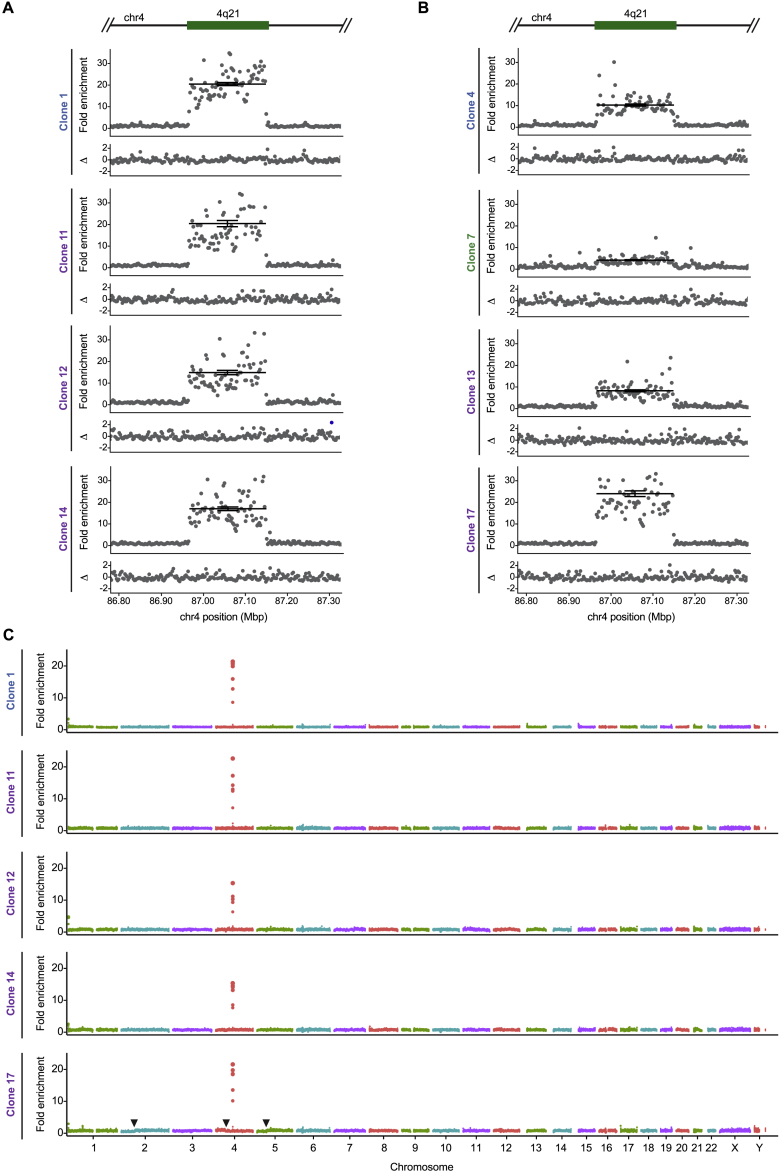
Copy-Number Variation (CNV) Analysis of 4q21 HAC Clones, Related to Figure 5 A,B) Fold-enrichment of the 4q21 and flanking sequences in the HAC-containing clones relative to parental cells (upper panels) and the difference of the deviation (lower panels). The copy number of the 4q21 sequence is increased 15-21-fold in clones that lack CENP-B, functional α-satellite, and additional sequences from host chromosomes relative to the endogenous locus (A). It is increased 4-24-fold in clones that lack CENP-B and functional α-satellite but have acquired additional sequences from host chromosomes relative to the endogenous locus (B). The average copy number of the 4q21 sequence within the HAC clones can be calculated using the following formula: FE x CN_P_, where FE is the average fold-enrichment of the 4q21 sequence in the HAC clone relative to the copy number at the endogenous locus and CN_P_ is the copy number of the 4q21 sequence in the parental cells (which is 2.7 on average, as determined by IF-FISH). The average copy number of the 4q21 sequence within the HACs in panel A is listed in Table S3. (C) Whole-genome CNV analysis in 4q21 HAC clones showing that the 4q21 sequence is selectively amplified in each cell line. Our CNV analysis can detect gross genome rearrangements, such as those in clone 17 where there is a reduction in copy number of the p-arm of chromosomes 2 and 5 and the q-arm of chromosome 4 (the points where the reduction begins is marked by an arrowhead). The genome rearrangements in this cell line may indicate that genome integrity was compromised in the cell that formed the HAC.

Our ChIP-seq studies revealed that the centromere on the four HACs essentially entirely comprised of 4q21 BAC^LacO^ vary widely in location (Figure 5A). CENP-A has maximal enrichment on different sequences within the construct, indicating that there is unlikely to be a small number of preferred sequences within the HAC that confer a propensity to establish functional centromeric chromatin. In two of the four clones (clones 11 and 14), the highest peaks of CENP-A enrichment are exclusively on the 4q21 genomic sequence, while on the other two (clones 1 and 12), the highest peaks also include the prokaryotic backbone of 4q21 BAC^LacO^ (Figure 5A). Using a genome-wide sequencing approach we recently applied to studies of centromere strength in mice (Iwata-Otsubo et al., 2017), we analyzed the total input mononucleosome populations isolated after micrococcal nuclease (MNase) digestion of chromatin (Figure S4) and found a substantial enrichment for sequences from chromosome 4q21 (Figure S5). Using established copy number variation analysis tools (Xie and Tammi, 2009), we found that each of the four HACs had substantially multimerized (multimerization varied from 41- to 55-fold, depending on the clone) (Figures 5A and S5; Table S3), consistent with the finding that these HACs exist as large entities in cells that are easily detectable by DAPI staining next to their natural counterparts (Figure 3C). This analysis also revealed that the sharp boundaries of CENP-A localization in some locations on the HACs (for instance in clone 11) (Figure 5A) are not due to amplifications of only specific regions of 4q21 BAC^LacO^ (Figure S4).

**Figure S6:**
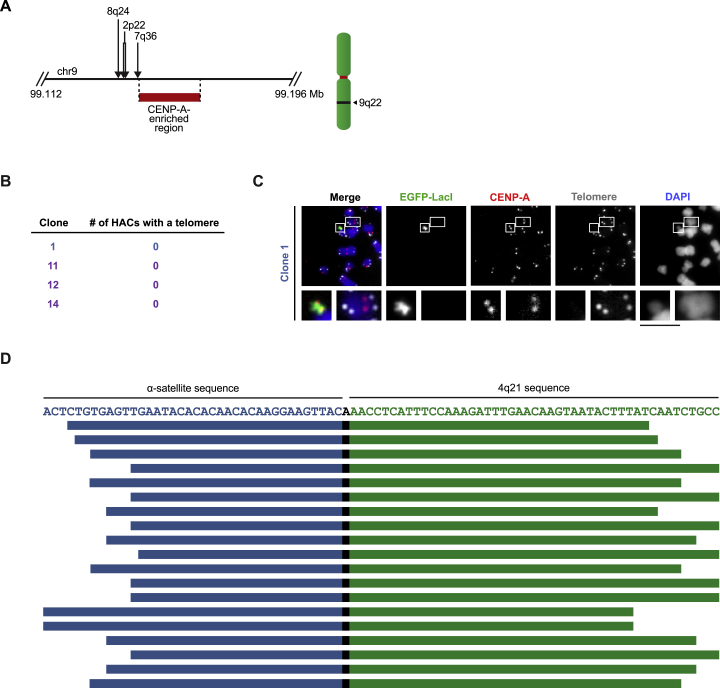
Analysis of the Genomic Alterations that Occur in Regions Flanking an Acquired Sequence, and Assessment of Telomere and Centromere Repeat Acquisition in Specific 4q21 BAC^LacO^ HACs, Related to Figure 5 (A) Chimeric reads (i.e., reads that contain sequences from more than one region in the genome) were found to map to the flanking regions of the CENP-A-enriched region in 9q22 in clone 17 as well as other regions in the genome (the location of the junction is shown with an arrow, and the secondary alignment position is listed above it). No chimeric reads mapped to the flanking regions of the CENP-A-enriched region of 3q13 in clone 17. Additionally, no chimeric reads spanning the 4q21 sequence and the flanking sequences of 3q13 or 9q22 were identified. These findings, together with our whole-genome CNV analysis results (Figure S5C), indicate that large-scale structural rearrangements likely occurred when the CENP-A-enriched region within chromosome 9q22 was acquired by the HAC. (B) Results of the telomere assay showing that telomeric repeats are not detected in 4q21 BAC^LacO^ HACs (n = 10, 11, 7, and 10, respectively). BAC-based HACs usually do not acquire telomeres, as reported for conventional HACs (Ebersole et al., 2000, Grimes et al., 2001). Although linear chromosomes need telomeres to buffer the loss of DNA sequences due to the end-replication problem, circular chromosomes, such as the HACs generated in this study as well as those found in nature, do not need these repeats to prevent gene loss. (C) Representative image of clone 1 showing that telomeric sequences are not detected at the HAC using a telomere repeat-specific FISH probe, unlike at endogenous chromosomes. The HAC is detected with EGFP-LacI, which binds the LacO repeats present in the HAC. Insets are 3.2× magnification. Bar, 10 μm. (D) Illustration of the 19 reads containing a junction between α-satellite and the 4q21 sequence within 4q21 BAC^LacO^ HAC clone 3. The sequence of the junction is shown (with α-satellite in blue and the 4q21 sequence in green), and the position and length of all 19 reads is indicated.

In contrast to the four HACs essentially entirely comprised of 4q21 BAC^LacO^, a clone (clone 13) that had acquired genomic sequences (Figure 4F) showed discreet CENP-A enrichment in several locations within 4q21 (Figure 5B). Additionally, it showed enrichment on acquired sequences that map to α-satellite DNA that normally does not harbor functional centromeric sequences (Figures 4D–4F; Table S4). Another clone (clone 17) had no strong sites of CENP-A enrichment within 4q21, but rather had acquired α-satellite DNA that normally does not harbor functional centromeric sequences (Figures 4D and 4F; Table S4) as well as two other non-centromeric sequences within the genome: one from 3q13 and another from 9q22 (Figure 5B; Table S4). This clone has apparent genomic rearrangements (Figure S5C), and deeper sequencing of the bulk nucleosome reads from this clone revealed several rearrangements not present in the parental cell line that are proximal to the 9q22 region incorporated into the HAC (Figure S6A). It seems likely, therefore, that genomic integrity was compromised in the cell that received 4q21 BAC^LacO^ and originated this particular HAC (clone 17). Together, our findings indicate that, unlike constructs containing α-satellite DNA, non-repetitive constructs can form HACs either directly (Figures 4F and 5A) or by acquiring one or several genomic sequences (Figures 4F and 5B) upon which functional centromeric chromatin is assembled to confer HAC establishment.

For the HACs that have a centromere that we can account for entirely with the 4q21 BAC^LacO^ sequences (clones 1, 11, 12, and 14) by our conventional ChIP-seq analysis (Figure 5A), we found that, like other prior HACs formed with circular constructs (Ebersole et al., 2000, Grimes et al., 2001), none of them had acquired telomeric sequences (Figures S6B and S6C). We devised a strategy to further probe these four clones for potential junctions with α-satellite sequences (Figure 5C). Our strategy employs the recently completed reference models of all autosomal and allosomal human centromeres (Miga et al., 2014) and searches for sequences in our ChIP-seq dataset containing k-mers for both 4q21 and any α-satellite DNA. In addition to the four HACs (clones 1, 11, 12, and 14), we included clone 3 that we found contained a small, but detectable, FISH signal for functional centromeric DNA (Figures 4D and S3B). We found that none of the four HACs we interrogated contains a single detectable junction with α-satellite DNA (Figure 5D) within the pool of >6,000,000 reads analyzed per HAC. Clone 3, however, contained 19 such junction reads (Figure 5D). These 19 reads vary in length on one or both ends of the read, but contain precisely the same junction site between 4q21 and a sequence that is from within a monomer of α-satellite DNA (Figure S6D). Indeed, this junction site maps to one side of the single strong peak of CENP-A nucleosome enrichment on 4q21 (Figure 5E). Thus, using an approach that is capable of readily identifying the presence of a junction and defines its site at single-nucleotide resolution, we failed to find any evidence of such junctions in the four HACs we identified that formed a centromere without acquiring genomic sequences.

**Figure 6:**
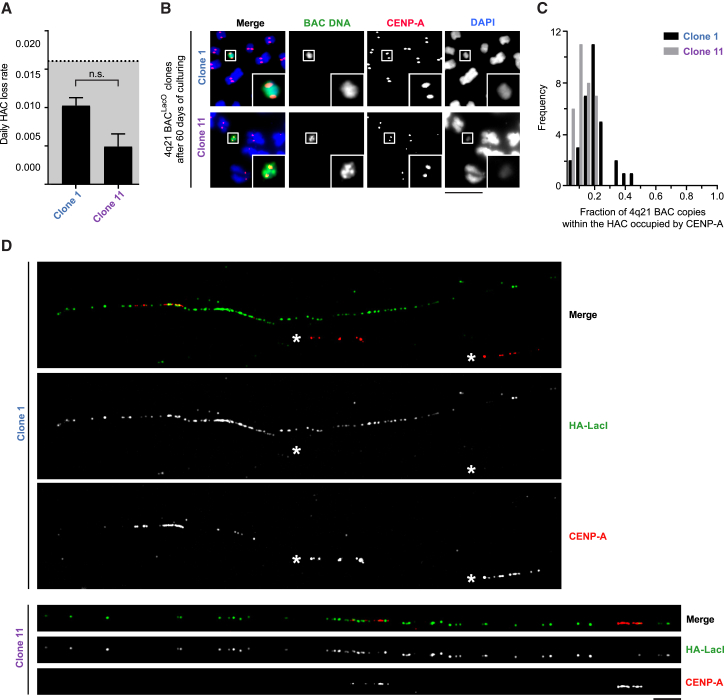
Stable 4q21 HACs that Have Not Acquired Genomic Sequences During Formation Harbor a Centromere with a High Local Density of CENP-A (A) Quantitation of the daily HAC loss rate in clones 1 and 11 after culturing without G418-S for 60 days (shading as in Figure 1H). The mean daily loss rate (± SEM) is shown. n = 120 cells pooled from 3 independent experiments for each clone. n.s., not significant. (B) Representative images of the HACs in clones 1 and 11 after 60 days of culturing in the absence of G418-S. Insets: 2.5× magnifications. Bar, 10 μm. (C) Histogram of the fraction of 4q21 BAC copies that are occupied by CENP-A within clones 1 and 11. 4q21 BAC copies were visualized on physically stretched chromatin fibers with immunodetection of CENP-A and expression of HA-LacI in cells. The 4q21 BAC DNA is represented by gaps between resolvable foci of HA-LacI. (D) Representative example of a stretched copy of the HAC in clones 1 and 11. CENP-A occupies discrete regions of neighboring copies of the 4q21 BAC^LacO^, spanning a similar cumulative distance of stretched chromatin as in neighboring centromeres in the same field (denoted by asterisks). Bar, 5 μm.

To investigate the stability and organization of HACs that have formed without acquiring genomic sequences, we focused our attention on detailed analysis of two clones, clones 1 and 11, that have clearly distinct CENP-A enrichment patterns from one another (Figure 5A). First, we found that the daily loss rate over 60 days in cell culture (Figures 6A and 6B) is similarly low for both HACs compared to the other HACs measured in this study (Figures 1H and 2J). Next, we sought to define the organization of each HAC with regard to their CENP-A occupancy in a way consistent with both their discrete paired sister centromere morphology (Figure 4C) and with the CENP-A ChIP-seq data mapped to the input 4q21 BAC^LacO^ sequence (Figure 5A). One possibility is that the sites of ChIP-seq enrichment represent low CENP-A occupancy per amplified copy of the 4q21 BAC^LacO^ that might coalesce in three dimensions on the HAC. Alternatively, only one or a small number of copies of 4q21 BAC^LacO^ house CENP-A, with the vast majority devoid of centromeric nucleosomes. To distinguish between these possibilities, we used stretched chromatin fibers (Blower et al., 2002, Iwata-Otsubo et al., 2017) to visualize HAC centromeres at high resolution (Figures 6C and 6D). We used HA-tagged LacI to identify the HAC and mark the portion of each copy of 4q21 BAC^LacO^ containing the LacO array (Figure 6D, visualized as a single green focus). For both clones 1 and 11, CENP-A largely occupies space on the fiber between these foci, consistent with our ChIP-seq mapping (Figure 5A), and only a small fraction of BACs (each copy of 4q21 BAC DNA is represented by a gap between LacO arrays) are occupied by CENP-A (Figures 6C and 6D). We also note that, for both clones, we observed several examples of fibers like the representative images shown where there is a major and minor site of CENP-A enrichment (closer together for clone 1 than for clone 11) (Figure 6D). Cumulatively, CENP-A nucleosomes occupy a region of high density similar to that on neighboring centromeres from endogenous chromosomes (Figure 6D; marked with an asterisk). Taking into consideration the 5.3–9.0 Mb size of the HACs (Table S3) and that our quantitation (Figure 6C) may somewhat overestimate the total fraction of the HAC occupied by CENP-A (see STAR Methods), we conclude that a typical copy of these HACs have regions of CENP-A that discontinuously span 0.5–1 Mb of neighboring copies of 4q21 BAC^LacO^. Further, we conclude that the major CENP-A ChIP-seq peaks (Figure 5A) each represent the position of CENP-A enrichment on one or a small number of individual copies of 4q21 BAC^LacO^.

Together, our mapping (Figures 5A and 5B), junction searching approaches (Figures 5C–5E), and chromosome stretching experiments (Figures 6C and 6D) highlight how HACs that bypass centromeric DNA allow for a comprehensive understanding of centromeric chromatin localization as well as HAC composition, organization, and copy number in a manner that is not imaginable with HACs built from centromeric DNA repeats.

## Discussion

Centromere formation on HACs has long been thought to require α-satellite DNA with a high density of CENP-B boxes (Ikeno et al., 1998, Ohzeki et al., 2002, Okada et al., 2007, Schueler et al., 2001), with proposals that a high density of local CENP-B on the naked DNA facilitates nearby nascent assembly of CENP-A nucleosomes (Okada et al., 2007) or stabilizes them upon formation (Fujita et al., 2015). Here, we report two other ways to establish a centromere during HAC formation. The first is a directed approach with local seeding of CENP-A nucleosomes on repetitive α-satellite DNA, taking advantage of a growing wealth of knowledge about the CENP-A nucleosome assembly pathway (Barnhart et al., 2011, Dunleavy et al., 2009, Foltz et al., 2009, Logsdon et al., 2015, Mendiburo et al., 2011). More surprisingly, however, we found that HACs can form on non-repetitive sequences without a requirement for seeding CENP-A nucleosomes, CENP-B boxes, or the expression of CENP-B, itself. Our functional tests with HACs are especially important to inform the further development of recent proposals for DNA sequence-based contributions to centromere identity (Kasinathan and Henikoff, 2018) and strength (Iwata-Otsubo et al., 2017).

The HACs we report that do not acquire α-satellite sequences during centromere formation (Figures 4F and 5A) are able to epigenetically maintain centromere identity in a manner we propose is analogous to non-repetitive neocentromeres in the human population (Alonso et al., 2003, Alonso et al., 2010, Hasson et al., 2011). Non-repetitive DNA that does not require seeding CENP-A nucleosome assembly might have been missed in previous HAC studies for several reasons. Sequences besides α-satellite were assumed to be implausible in human cells because of the reported CENP-B requirement (Ohzeki et al., 2002, Okada et al., 2007), the failure of α-satellite DNA with a low density of CENP-B boxes (Schueler et al., 2001), and the failure of two different non-repetitive genomic sequences (from chromosomes 10, Saffery et al., 2001; and X, Grimes et al., 2002; respectively). Indeed, HAC formation on 4q21 BAC^LacO^ is relatively rare, occurring in only one or a few clones isolated in a typical HAC experiment (Figure 3). Thus, previous conclusions likely precluded the exploration of non-repetitive sequences.

**Figure S7:**
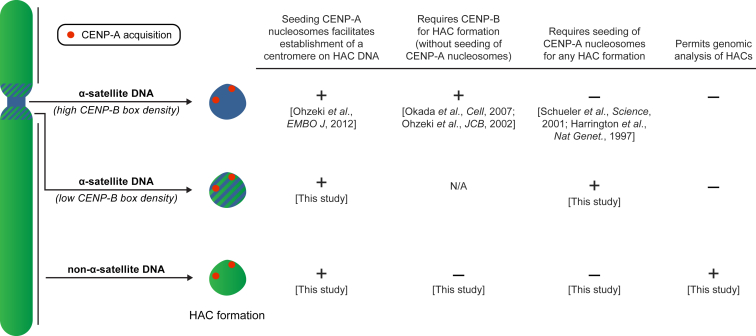
Three Types of DNA Sequences Are Competent for Centromere Formation on a HAC, Related to Figure 7 and Discussion See the Discussion section of the main text for details.

**Figure 7:**
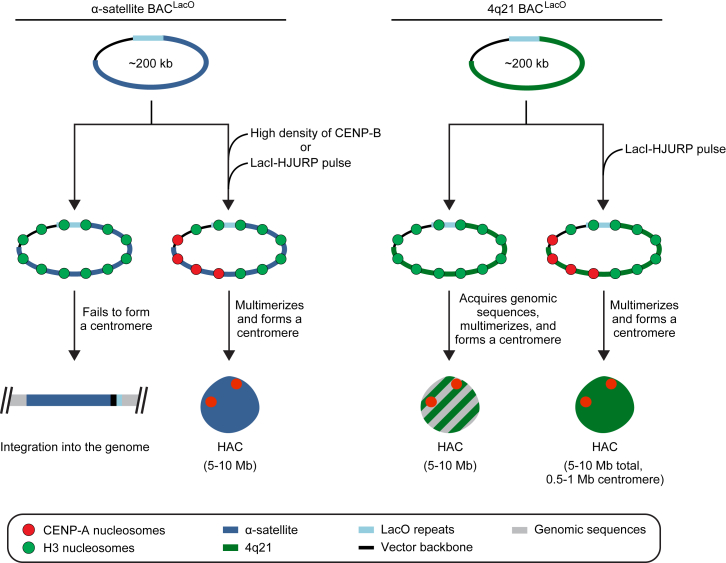
Pathways to HAC Formation Cartoon drawing summarizing the findings in this study. In the absence of a high density of CENP-B binding or CENP-A nucleosome seeding, the α-satellite BAC^LacO^ vectors fail to form a centromere and are subsequently integrated into the genome. Alternatively, when α-satellite BAC^LacO^ vectors have a high density of CENP-B binding or are epigenetically seeded with CENP-A nucleosomes via LacI-HJURP, they assemble centromeric chromatin, multimerize, and form a functional HAC. When non-α-satellite vectors such as 4q21 BAC^LacO^ are introduced to human cells, they also can integrate into the genome (not depicted here), but strikingly can form HACs without CENP-B boxes or seeding of centromeric nucleosome assembly. In the absence of CENP-A nucleosome seeding via LacI-HJURP, 4q21 BAC^LacO^ vectors acquire host genomic sequences, which impart centromere competency to the vector and lead to the formation of a HAC. These sequences often consist of both α-satellite and non-α-satellite from host chromosomes. However, when CENP-A nucleosome assembly is directly seeded onto the 4q21 BAC^LacO^ vectors, centromeric chromatin is assembled onto the vector, it multimerizes, and a HAC is formed. The CENP-A nucleosomes reside on a small fraction of the 4q21 BAC^LacO^ vector sequences on the HAC, taking up the equivalent of 0.5–1.0 Mb of sequence, which is similar to the size observed at normal, endogenous human centromeres. See also Figure S7.

We propose a model wherein there are three types of human genomic sequences that can form a HAC (Figure S7). The first two are α-satellite DNA with either a high or low density of functional CENP-B boxes. We envision that both types of α-satellite DNA are similar in their inherent resistance to initial CENP-A nucleosome assembly and/or resistance to establishing a self-propagating centromere because they are susceptible to CENP-A nucleosome displacement by invading heterochromatin. The resistance can be overcome by either a high density of CENP-B binding (Figure 7) (Nakano et al., 2008, Schueler et al., 2001) and accelerated further by CENP-A overexpression (Pesenti et al., 2018) or local targeting of CENP-A nucleosome assembly with HJURP (Figure 7). Once the initial resistance is overcome, the natural epigenetic centromere propagation mechanism takes hold, wherein the high local concentration of existing CENP-A nucleosomes directs the formation of nascent CENP-A nucleosomes on nearby DNA (Black and Cleveland, 2011, McKinley and Cheeseman, 2016). On first blush, our proposal that the sequences found at all normal human centromeres would be inherently resistant to centromere formation seems paradoxical. One must remember, though, that centromere movement is very slow relative to the timescale of cell divisions. Epialleles of CENP-A location within a “sea” of highly repetitive DNA (most of which is packaged by canonical nucleosomes) are reported to be heritable through the germline (Maloney et al., 2012). Taking our findings into account, centromeres appear to evolve to restrict the pace of movement of CENP-A-containing chromatin, providing a potential explanation to the paradox of why α-satellite DNA is found at all normal human centromeres even though it is not required for centromere identity and function (Eichler, 1999). If α-satellite repeats were inherently neutral or permissive to new centromere formation, then the high local density of CENP-A nucleosomes generated by its self-templated mechanism for propagation might be compromised by the rapid attraction to any of the other α-satellite repeats at a given centromere. Rather, once established, centromeres are built to be stable chromatin domains.

The third type of sequence that can form a HAC lacks repetitive DNA or any CENP-B boxes but is competent for centromere formation (Figure S7). When centromere formation occurs on 4q21 BAC^LacO^ via seeding CENP-A nucleosome assembly, the HACs frequently form without acquiring other DNA sequences (Figure 7). Without seeding of CENP-A nucleosomes, HACs still form, albeit through acquiring CENP-B-positive α-satellite, CENP-B-negative α-satellite, or mixtures of genomic sequences including regions where new centromeres form on non-repetitive sequences (Figure 7). This DNA acquisition pathway to centromere formation was never observed with our α-satellite-based HACs. Thus, the sequence of input naked DNA HAC templates strongly impacts and extends the possible routes to centromere formation and potential ultimate success in generating functional HACs.

In terms of the overall outlook for HACs, our findings reveal surprising flexibility in how one can form centromeres in mammalian cells, indicating that it is possible to surmount two major limitations of HACs (low formation efficiency as shown in Basu and Willard, 2005 and the strict requirement for including substantial amounts of highly repetitive α-satellite DNA as shown in Grimes et al., 2002, Ikeno et al., 1998, and Ohzeki et al., 2002). First, HAC formation on α-satellite constructs can be substantially increased by a single pulse of epigenetic seeding of centromeric chromatin assembly. Strategies to increase HAC formation efficiency are desperately needed in order to achieve their potential in synthetic biology, especially if they are to serve as the basis for entirely synthetic chromosomes (Boeke et al., 2016). Second, HAC formation can occur without α-satellite DNA, and, when coupled to initial seeding of CENP-A nucleosome, frequently does so without acquiring other genomic sequences. With our methodology to circumvent the prior absolute requirement for CENP-B boxes/CENP-B protein (Ohzeki et al., 2002, Okada et al., 2007) and repetitive sequences, one could now envision simplifying schemes to make completely synthetic HAC templates (Nakano et al., 2008).

Prior studies have established that a typical route for HAC formation includes either simple or complex rearrangements that culminate in reassembly of a continuous molecule >1 Mb in size (Kouprina et al., 2012). The HACs in this study that underwent our full genomic analysis are each in the 5–10 Mb range and appear to have been formed with similar rearrangements and reassembly (Figures S3C and S3D). For the 4q21 BAC^LacO^ HACs, what stands out is their ability to form without detectable acquisition of genomic DNA, but only when there is prior seeding of CENP-A nucleosomes by HJURP targeting. This targeting event likely extends from the moment of introduction of BAC^LacO^ constructs through several initial cell cycles. LacO-tethered HJURP likely acts to both support nascent CENP-A nucleosome assembly and ward off heterochromatin (as has been reported upon cellular introduction of ectopic α-satellite DNA; Ohzeki et al., 2012), providing the time it takes for a self-propagating epigenetic centromere to form. Later, after clonal HACs are isolated, CENP-A nucleosome seeding by HJURP targeting is no longer necessary for the centromere on the HAC. The HAC is now propagated indistinguishably from other centromeres. The fact that non-repetitive DNA can act as a self-propagating centromere is central to our current understanding of the epigenetic underpinnings of centromere specification (i.e., what are termed neocentromeres, *de novo* centromeres, and/or evolutionary new centromeres; Fu et al., 2013, Liu et al., 2015, Montefalcone et al., 1999, Schneider et al., 2016, Shang et al., 2010, Tolomeo et al., 2017, Topp et al., 2009, Wade et al., 2009, Wang et al., 2014). The strategy we report in this paper provides a way to make a new centromere in the context of an artificial chromosome that should be widely applicable among mammals and more broadly to many eukaryotic systems.

Combining the methodologies from synthetic chromosome efforts in yeast with new innovations in HACs presents an attractive avenue for synthetic biology efforts. The development of tools and automation to synthesize and analyze yeast chromosomes should now be extended to mammalian systems to accelerate HAC development. For instance, HAC studies are currently slowed by the requirement of clonal isolation of many cell lines (e.g., 453 cell lines were isolated for this study alone) and very low-throughput analysis tools (i.e., combined IF-FISH to identify HAC-containing lines), all of which would benefit from streamlined methodologies and emerging instrumentation. Our study reveals that one promising avenue will be to develop non-repetitive HAC vectors that will allow annotation of copy number and organization of the functional centromere on every isolated clone. Centromeric α-satellite DNA is the most abundant highly repetitive DNA in humans, constituting ∼3% of our genome (Miga, 2017). Its repetitive nature has substantially slowed progress in HAC development because it is difficult to synthesize, a major challenge to clone and amplify without unwanted recombination, and refractory to characterization using genomic approaches. Our study reveals molecular requirements for centromere establishment and demonstrates that α-satellite DNA can be bypassed altogether, thereby greatly facilitating the construction of HACs and expanding the toolbox for centromere biology studies, gene therapy applications, and synthetic biology efforts.

## STAR★Methods

### Key Resources Table

**Table:** 

REAGENT or RESOURCE	SOURCE	IDENTIFIER
**Antibodies**		

Mouse monoclonal anti-CENP-A	Enzo	Cat# ADI-KAM-CC006-E
Mouse monoclonal anti-CENP-A	Abcam	Cat# ab13939
Mouse monoclonal anti-α-tubulin	Sigma-Aldrich	Cat# T9026
Mouse anti-CENP-B ascites	D. Cleveland; Earnshaw et al., 1987	2D-7
Rabbit polyclonal anti-CENP-B	Santa Cruz Biotechnology	Cat# sc-22788
Rabbit monoclonal anti-HA	Cell Signaling Technology	Cat# 3724S
Rabbit polyclonal anti-GFP	Logsdon et al., 2015	N/A
Rabbit anti-HJURP	Bassett et al., 2012	N/A
Human anti-centromere (ACA)	Antibodies Incorporated	Cat# 15-235
Cy3-conjugated donkey anti-mouse	Jackson ImmunoResearch	Cat# 715-165-151
Cy3-conjugated goat anti-rabbit	Jackson ImmunoResearch	Cat# 111-165-144
Cy5-conjugated donkey anti-mouse	Jackson ImmunoResearch	Cat# 715-175-151
Cy5-conjugated donkey anti-rabbit	Jackson ImmunoResearch	Cat# 711-175-152
FITC-conjugated goat anti-rabbit	Jackson ImmunoResearch	Cat# 111-095-144
Neutravidin-FITC	Thermo Fisher Scientific	Cat# 31006
HRP-conjugated donkey anti-rabbit	GE Healthcare	Cat# NA934V
HRP-conjugated donkey anti-mouse	GE Healthcare	Cat# NA931V
HRP-conjugated donkey anti-human	Jackson ImmunoResearch	Cat# 109-035-149

**Bacterial and Virus Strains**		

*Escherichia coli*: ElectroMAX DH10B cells	Thermo Fisher Scientific	Cat# 18290-015
*Escherichia coli*: HB101 cells	Thermo Fisher Scientific	Cat# 18296-012

**Chemicals, Peptides, and Recombinant Proteins**		

FISH probe: TelC-Cy3	PNA Bio	Cat# P1002

**Critical Commercial Assays**		

Nick Translation Kit	Roche	Cat# 10976776001

**Deposited Data**		

Raw NGS data for 4q21 HAC clones 1, 4, 7, and 11-17	This paper	BioProject: PRJNA487691

**Experimental Models: Cell Lines**		

Human: 293GP cells	D. Cleveland; Morgenstern and Land, 1990	N/A
Human: HT1080 HILO RMCE acceptor cells	E. Makeyev; Khandelia et al., 2011	N/A
Human: HT1080^Dox-inducible mCherry-LacI-HJURP^ cells	This paper	N/A
Human: HT1080^Dox-inducible mCherry-LacI-HJURP^ CENP-B KO cells	This paper	N/A
Human: HT1080^Dox-inducible mCherry-LacI-HJURP^ chr11 α-satellite HAC CENP-B KO cells	This paper	BB978, CBKO, +Dox, C. 7
Human: HT1080^Dox-inducible mCherry-LacI-HJURP^ chr11 α-satellite HAC mCherry-LacI-HJURP KO cells	This paper	BB978, mChKO, +Dox, C. 7
Human: HAC Clone 1	This paper	BB1067, +Dox, C. 5
Human: HAC Clone 2	This paper	BB1067, +Dox, C. 9
Human: HAC Clone 3	This paper	BB1067, -Dox, C. 7
Human: HAC Clone 4	This paper	BB1067, -Dox, C. 15
Human: HAC Clone 5	This paper	BB1067, -Dox, C. 16
Human: HAC Clone 6	This paper	BB1067, -Dox, C. 9
Human: HAC Clone 7	This paper	BB1207, n1, C. 8
Human: HAC Clone 8	This paper	BB1207, n1, C. 12
Human: HAC Clone 9	This paper	BB1207, n2, C. 4
Human: HAC Clone 10	This paper	BB1207, n3, C. 8
Human: HAC Clone 11	This paper	BB1067, CBKO, +Dox, C. 1
Human: HAC Clone 12	This paper	BB1067, CBKO, +Dox, C. 3
Human: HAC Clone 13	This paper	BB1067, CBKO, +Dox, C. 4
Human: HAC Clone 14	This paper	BB1067, CBKO, +Dox, C. 5
Human: HAC Clone 15	This paper	BB1067, CBKO, +Dox, C. 12
Human: HAC Clone 16	This paper	BB1067, CBKO, -Dox, C. 15
Human: HAC Clone 17	This paper	BB1067, CBKO, -Dox, C. 10
Human: HAC Clone 18	This paper	BB977, +Dox, n1, C. 1
Human: HAC Clone 19	This paper	BB977, +Dox, n1, C. 3
Human: HAC Clone 20	This paper	BB977, +Dox, n1, C. 14
Human: HAC Clone 21	This paper	BB977, +Dox, n1, C. 17
Human: HAC Clone 22	This paper	BB977, +Dox, n1, C. 19
Human: HAC Clone 23	This paper	BB978, +Dox, n2, C. 2
Human: HAC Clone 24	This paper	BB978, +Dox, n2, C. 7
Human: HAC Clone 25	This paper	BB978, +Dox, n2, C. 12
Human: HAC Clone 26	This paper	BB978, +Dox, n2, C. 14
Human: HAC Clone 27	This paper	BB978, +Dox, n2, C. 15
Human: HAC Clone 28	This paper	BB978, +Dox, n2, C. 20
Human: HAC Clone 29	This paper	BB978, mChKO, +Dox, C. 6
Human: HAC Clone 30	This paper	BB978, mChKO, +Dox, C. 9
Human: HAC Clone 31	This paper	BB978, mChKO, +Dox, C. 12
Human: HAC Clone 32	This paper	BB978, CBKO, +Dox, C. 2
Human: HAC Clone 33	This paper	BB978, CBKO, +Dox, C. 4
Human: HAC Clone 34	This paper	BB978, CBKO, +Dox, C. 5
Human: HAC Clone 35	This paper	BB978, CBKO, +Dox, C. 8
Human: HAC Clone 36	This paper	BB978, CBKO, +Dox, C. 9
Human: HAC Clone 37	This paper	BB978, CBKO, +Dox, C. 13
Human: HAC Clone 38	This paper	BB978, CBKO, +Dox, C. 20
Human: HAC Clone 39	This paper	BB978, CBKO, +Dox, C. 7
Human: HAC Clone 40	This paper	BB978, CBKO, +Dox, C. 11
Human: HAC Clone 41	This paper	BB978, CBKO mono, +Dox, C. 2
Human: HAC Clone 42	This paper	BB978, CBKO mono, +Dox, C. 3
Human: HAC Clone 43	This paper	BB978, CBKO mono, +Dox, C. 6

**Oligonucleotides**		

CENP-B sgRNA oligo 1 sequence: 5′- CACCGgaagaacaagcgcgccatcc-3′	This paper	N/A
CENP-B sgRNA oligo 2 sequence: 5′- AAACggatggcgcgcttgttcttcC-3′	This paper	N/A
mCherry sgRNA oligo 1 sequence: 5′-CACCGctcgaactcgtggccgttca-3′	This paper	N/A
mCherry sgRNA oligo 2 sequence: 5′-AAACtgaacggccacgagttcgagC-3′	This paper	N/A
BAC primer set 1 oligo 1 sequence: 5′- ttaatgaattacaacagtactgcgatgagtggcagg-3′	This paper	N/A
BAC primer set 1 oligo 2 sequence: 5′-gagcaatatagtcctacaatgtcaagctcga-3′	This paper	N/A
BAC primer set 2 oligo 1 sequence: 5′- ttaatgaattacaacagtactgcgatgagtggcagg-3′	This paper	N/A
BAC primer set 2 oligo 2 sequence: 5′- tcgaaggccctagtgtgctggaattcgcccttactg-3′	This paper	N/A
LacO primer 1: 5′-agtggacatttcgaccacat-3′	This paper	N/A
LacO primer 2: 5′-atttttatgggccacaaatt-3′	This paper	N/A

**Recombinant DNA**		

Plasmid: pEM791	E. Makeyev; Khandelia et al., 2011	N/A
Plasmid: pEM784	E. Makeyev; Khandelia et al., 2011	N/A
Plasmid: mCherry-LacI-HJURP	D. Foltz; Barnhart et al., 2011	N/A
Plasmid: pLacO	Mendiburo et al., 2011	N/A
Plasmid: pEGFP-C1	Clontech	N/A
Plasmid: pcDNA5-FRT-TO-H2B-AID-YFP	D. Cleveland; Holland et al., 2012	N/A
Plasmid: lentiCRISPR v2	F. Zhang	Addgene 52961
Plasmid: pMD2.G	Addgene	Addgene 12259
Plasmid: psPax2	Addgene	Addgene 12260
Plasmid: pcDNA3.1-HA-LacI-CENP-A	Logsdon et al., 2015	BB695
Plasmid: EGFP-AID-CENP-A	Fachinetti et al., 2017	BB1051
Plasmid: pLacO_KanR_NeoR	This paper	BB735
Plasmid: pN2-LexA-FLAG	This paper	BB694
Plasmid: pLacO_KanR_NeoR + LoxP511 + 4x LexA-binding sites	This paper	BB975
Plasmid: Doxycycline-inducible mCherry-LacI-HJURP	This paper	BB730
Plasmid (lentiviral): CENP-B sgRNA / Cas9-P2A-HygR	This paper	BB1117
Plasmid (lentiviral): mCherry sgRNA / Cas9-P2A-HygR	This paper	BB1131
Plasmid (lentiviral): HA-LacI	This paper	BB1174
Plasmid (lentiviral): EGFP-LacI	This paper	BB1175
BAC: chr7 α-sat BAC	BACPAC Resources Center	RP11-435D24
BAC: chr11 α-sat BAC	BACPAC Resources Center	RP11-100E23
BAC: 4q21 BAC	BACPAC Resources Center	RP11-1064O23
BAC: 4q22 BAC	BACPAC Resources Center	RP11-141P18
BAC: 4q28 BAC	BACPAC Resources Center	RP11-142K9
BAC: 8q21 BAC	BACPAC Resources Center	RP11-90G23
BAC: chr7 α-sat BAC^LacO^	This paper	BB977
BAC: chr11 α-sat BAC^LacO^	This paper	BB978
BAC: 4q21 BAC^LacO^	This paper	BB1067
BAC: 4q22 BAC^LacO^	This paper	BB1066
BAC: 4q28 BAC^LacO^	This paper	BB976
BAC: 8q21 BAC^LacO^	This paper	BB1065

**Software and Algorithms**		

ImageQuant 400	GE Healthcare	N/A
ImageJ	Schneider et al., 2012	https://imagej.nih.gov/ij/
CRaQ	Bodor et al., 2012	http://facilities.igc.gulbenkian.pt/microscopy/macros/CRaQ_v1.12.ijm
Bowtie 2	Langmead and Salzberg, 2012	http://bowtie-bio.sourceforge.net/bowtie2/index.shtml
cutadapt	Martin, 2011	https://cutadapt.readthedocs.io/en/stable/
SAMtools	Li et al., 2009	http://samtools.sourceforge.net/
BEDtools	Quinlan and Hall, 2010	https://bedtools.readthedocs.io/en/latest/
deepTools	Ramírez et al., 2014	https://deeptools.readthedocs.io/en/develop/
BWA-MEM	Li, 2013, Li and Durbin, 2010	http://bio-bwa.sourceforge.net/
GraphPad Prism	GraphPad Software	https://www.graphpad.com
CNV-Seq	Xie and Tammi, 2009	http://tiger.dbs.nus.edu.sg/cnv-seq/
BLASTn	Altschul et al., 1990	https://blast.ncbi.nlm.nih.gov/Blast.cgi?PAGE_TYPE=BlastSearch

### Lead Contact and Materials Availability

Further information and requests for resources and reagents should be directed to and will be fulfilled by the Lead Contact, Ben E. Black (blackbe@pennmedicine.upenn.edu).

### Experimental Model and Subject Details

#### Cell lines

Human HT1080 HILO RMCE accepter cells (Khandelia et al., 2011) (male fibrosarcoma; a gift from E. Makeyev, Nanyang Technological University, Singapore) and derivative cell lines were cultured in DMEM supplemented with 10% FBS, 100 U/mL penicillin, and 100 μg/mL streptomycin. HT1080^Dox-inducible mCherry-LacI-HJURP^ cells were supplemented with 2 μg/mL puromycin, and CENP-B knockout or mCherry knockout HT1080^Dox-inducible mCherry-LacI-HJURP^ cells were supplemented with 2 μg/mL puromycin and 400 μg/mL hygromycin. HT1080^Dox-inducible mCherry-LacI-HJURP^ cells containing a HAC were supplemented with 800 μg/mL G418-S. All HT1080 cell lines were maintained at 37°C in a humidified incubator with 5% CO_2_. HT1080 HILO RMCE acceptor cells and HT1080^Dox-inducible mCherry-LacI-HJURP^ cells were authenticated via short tandem repeat (STR) allele analysis by Duke University DNA Analysis Facility.

The HT1080^Dox-inducible mCherry-LacI-HJURP^ cell line was generated via recombinase-mediated cassette exchange (RMCE) using the HILO RMCE system (Khandelia et al., 2011). This system allowed us to insert the dox-inducible mCherry-LacI-HJURP transgene cassette at a single genomic locus. Briefly, a monoclonal HT1080 accepter cell line with *loxP* and *lox2272* recombination sites at a single chromosomal locus was co-transfected with a donor plasmid containing an mCherry-LacI-HJURP gene under the control of a tetracycline-responsive element (TRE) (all flanked by *loxP* and *lox2272* sites) and a second plasmid expressing Cre recombinase (pEM784; Khandelia et al., 2011). The cells were co-transfected at a 100:1 ratio (990 ng mCherry-LacI-HJURP donor plasmid and 10 ng Cre recombinase plasmid) using FuGENE 6 (Promega). 2 days after transfection, 2 μg/mL puromycin was added to the growth medium for selection of the stable cell line. 2 μg/mL doxycycline was added to the growth medium for 24 h to induce expression of mCherry-LacI-HJURP from the TRE promoter.

The CENP-B knockout HT1080^Dox-inducible mCherry-LacI-HJURP^ cell line was generated by lentiviral delivery of a transgene expressing a CENP-B-specific sgRNA and Cas9-P2A-HygR. Briefly, HT1080^Dox-inducible mCherry-LacI-HJURP^ cells were plated into a single well of a 6-well plate and allowed to adhere to the bottom of the plate. The next day (when cells were ∼70% confluent), 50 μL of viral supernatant was added to the culture. The cells were split into a 10 cm plate 24 h later, and 400 μg/mL hygromycin was added after an additional 24 h (48 h post-transduction). Cells were maintained in 400 μg/mL hygromycin and 2 μg/mL puromycin during selection. Monoclonal lines were isolated, expanded, and screened by immunofluorescence and immunoblot to identify a cell line that had CENP-B knocked out and expressed mCherry-LacI-HJURP to a similar level as the wild-type parental cell line. Genomic PCR and sequencing were performed to verify the presence of an indel at the expected region within the CENP-B gene.

The CENP-B knockout and mCherry-LacI-HJURP knockout chr11 α-satellite HAC HT1080^Dox-inducible mCherry-LacI-HJURP^ monoclonal cell lines were generated by lentiviral delivery of a plasmid expressing either a CENP-B-specific or mCherry-specific sgRNA and Cas9-P2A-HygR to HT1080^Dox-inducible mCherry-LacI-HJURP^ cells harboring a chr11 α-satellite HAC. By targeting Cas9 to the CENP-B or mCherry genes, a double-stranded break is generated within the DNA-binding domain or β-barrel of CENP-B and mCherry genes, respectively, which, when repaired via NHEJ, generates an indel that leads to a premature stop codon. Briefly, HT1080^Dox-inducible mCherry-LacI-HJURP^ cells containing a chr11 α-satellite HAC were plated into a single well of a 6-well plate (in the presence of 800 μg/mL G418-S) and allowed to adhere to the bottom of the plate. The next day (when cells were ∼70% confluent), 5 or 50 μL of viral supernatant was added to the culture in the presence of 8 μg polybrene (Specialty Media, TR-1003-G). The cells were split into a 10 cm plate 24 h later (in media supplemented with 800 μg/mL G418-S), and 400 μg/mL hygromycin was added after an additional 24 h (48 h post-transduction). Cells were maintained in 800 μg/mL G418-S and 400 μg/mL hygromycin during selection. Monoclonal lines were isolated, expanded, and screened by immunofluorescence, immunoblot, and combined immunofluorescence-fluorescence *in situ* hybridization (IF-FISH) to identify three monoclonal cell lines that had either CENP-B or mCherry-LacI-HJURP knocked out and also contained a chr11 α-satellite HAC in a proportion of cells. Genomic PCR and sequencing were performed to verify the presence of a translation-disrupting indel at the expected region within the CENP-B or mCherry genes.

### Method Details

#### Plasmid construction

The doxycycline-inducible mCherry-LacI-HJURP donor plasmid (used to make the HT1080^Dox-inducible mCherry-LacI-HJURP^ cell line via RMCE) was constructed by digesting a plasmid containing mCherry-LacI-HJURP (Barnhart et al., 2011) (a gift from D. Foltz, Northwestern University) with PsiI and AgeI-HF to isolate the mCherry-LacI-HJURP gene. A donor plasmid containing a TRE floxed by *loxP* and *lox2272* recombination sites (Khandelia et al., 2011) (pEM791; a gift from E. Makeyev, Nanyang Technological University, Singapore) was digested with BsrGI, blunted with Klenow DNA polymerase (NEB), and then digested with AgeI-HF. The mCherry-LacI-HJURP fragment was ligated to the pEM791 backbone to produce the doxycycline-inducible mCherry-LacI-HJURP donor plasmid.

The LacO plasmid used in the Cre-Lox recombination reactions was constructed by digesting a LacO-containing plasmid (Mendiburo et al., 2011) with AseI and XhoI to isolate the LacO repeats, digesting pEGFP-C1 (Clontech) with AseI and SalI to isolate the Kan^R^/Neo^R^-containing backbone, and ligating the two fragments together to generate pLacO_KanR_NeoR. Then, 4x LexA-binding sites and a *loxP511* site were PCR-amplified from a derivative of pN2-LexA-FLAG. The PCR fragment and pLacO_KanR_NeoR were digested with EcoO1091 and ligated together to generate the LacO plasmid containing 4x LexA-binding sites, a *loxP511* site, and Kan^R^/Neo^R^. LacO-containing plasmids were propagated in HB101 cells (a *recA-* strain) and grown at 30°C to prevent recombination and subsequent loss of the LacO repeats. All plasmids were verified by restriction digest and sequencing.

The CENP-B sgRNA / Cas9-P2A-HygR lentiviral plasmid (used to knock out CENP-B in HT1080 cells) was constructed by annealing oligos containing the CENP-B targeting sequence (Fachinetti et al., 2015) and sticky ends from a BsmBI site (5′- CACCGgaagaacaagcgcgccatcc-3′ and 5′- AAACggatggcgcgcttgttcttcc-3′; the CENP-B targeting sequence is lowercase, and the BsmBI sticky ends are uppercase) and ligating the annealed oligos into a lentiCRISPR v2 plasmid [a gift from F. Zhang, MIT (Addgene plasmid #52961)] backbone that had been digested with BsmBI. The resulting vector and a gBlock containing a BamHI-P2A-XhoI-BsrGI-WPRE-SacII sequence were digested with BamHI and SacII and ligated together to allow the Puro^R^ gene to be swapped out with a Hyg^R^ gene. Then, the Hyg^R^ gene was PCR-amplified from pcDNA5-FRT-TO-H2B-AID-YFP (a gift from D. Cleveland, UCSD), digested with XhoI and BsrGI, and ligated into the plasmid at the same restriction sites, resulting in a lentiCRISPR v2 plasmid containing a CENP-B sgRNA and Cas9-P2A-HygR.

The mCherry sgRNA / Cas9-P2A-HygR lentiviral plasmid (used to knock out mCherry-LacI-HJURP in HT1080 cells) was constructed by annealing oligos containing the mCherry targeting sequence (Carlson-Stevermer et al., 2016) and sticky ends from a BsmBI site (5′-CACCGctcgaactcgtggccgttca-3′ and 5′-AAACtgaacggccacgagttcgagC-3′; the mCherry targeting sequence is lowercase, and the BsmBI sticky ends are uppercase) and ligating the annealed oligos into a lentiCRISPR v2 plasmid backbone that had been digested with BsmBI. The resulting vector and the CENP-B sgRNA / Cas9-P2A-HygR lentiviral plasmid (described above) were digested with NotI-HF and NheI and ligated together to generate a lentiCRISPR v2 plasmid containing an mCherry sgRNA and Cas9-P2A-HygR.

The HA-LacI lentiviral plasmid (used to generate HA-LacI lentivirus for detection of the BAC^LacO^ constructs) was constructed by PCR-amplifying the HA-LacI gene from a derivative of pcDNA3.1, pcDNA3.1-HA-LacI-CENP-A (Logsdon et al., 2015). The resulting PCR fragment was digested with BsrGI and XhoI and ligated into the backbone of a derivative lentiCRISPR v2 that had been digested with Acc65I and XhoI. This generated a lentiCRISPR v2 plasmid containing the HA-LacI gene in place of the Cas9 gene.

The EGFP-LacI lentiviral plasmid (used to generate EGFP-LacI lentivirus for detection of the BAC^LacO^ constructs) was constructed by PCR-amplifying the EGFP gene from EGFP-AID-CENP-A (Fachinetti et al., 2017). The resulting PCR fragment was digested with KpnI and EcoRI-HF and ligated into the backbone of the HA-LacI lentiviral plasmid that had been digested with the same enzymes. This generated a lentiCRISPR v2 plasmid containing the EGFP-LacI gene in place of the Cas9 gene. All lentiviral vectors were verified by sequencing.

#### Cre-Lox recombination of BACs

Available BACs were purchased from BACPAC Resources Center (BPRC) at the Children’s Hospital Oakland Research Institute (CHORI). Given name, clone name, and NCBI clone ID are as follows: chr7 α-sat (RP11-435D24; 560470), chr11 α-sat (RP11-100E23; 204304), 4q21 (RP11-1064O23; 451609), 4q22 (RP11-141P18; 217787), 4q28 (RP11-142K9; 218037), and 8q21 (RP11-90G23; 257814). The neocentromere-proximal BAC clones were chosen because they were located within 150 kbp of the neocentromere (as defined by Hasson et al., 2013) and were 150-190 kbp in length, similar to the α-satellite BACs.

All BACs were prepped using PureLink HiPure Plasmid Midiprep Kit (Thermo Fisher Scientific) with protocol modifications for BACs. The BACs were checked by restriction digest and PFGE to ensure they were the expected sizes prior to performing the Cre-Lox recombination reaction. To recombineer the BACs, the LacO plasmid was digested with HindIII-HF to remove the pUC origin. By removing the pUC origin, the LacO plasmid is unable to propagate in bacteria cells in the event that it does not recombine with the BAC. The digested backbone was isolated and re-circularized via a ligation reaction and purified using a PCR purification kit (QIAGEN). The BAC and the LacO plasmid lacking the pUC origin were mixed in a 1:1 molar ratio (700-800 ng BAC DNA and ∼60 ng LacO plasmid without origin) with Cre recombinase (NEB; M0298) such that there was 1 U Cre for every 106.26 fmol of BAC DNA (based on NEB’s protocol). The reactions were incubated at 37°C for 30 min and then heat-inactivated at 70°C for 10 min. Reactions were ethanol-precipitated and electroporated in ElectroMAX DH10B cells (Thermo Fisher Scientific; 18290015) using a 0.1 cm cuvette at 2.0 kV, 200 Ω, and 25 μF. Electroporated cells were recovered at 30°C, plated onto LB plates supplemented with chloramphenicol (12.5 μg/mL) and kanamycin (25 μg/mL), and incubated at 30°C. Bacterial colonies were screened by colony PCR using two sets of primers [Primer set 1: 5′- ttaatgaattacaacagtactgcgatgagtggcagg-3′ and 5′-gagcaatatagtcctacaatgtcaagctcga-3′ (amplifies sequences within the BAC); Primer set 2: 5′- ttaatgaattacaacagtactgcgatgagtggcagg-3′ and 5′-tcgaaggccctagtgtgctggaattcgcccttactg-3′ (amplifies sequences within BAC^LacO^)] to confirm the incorporation of the LacO plasmid into the BAC. BAC^LacO^ constructs were validated by restriction digest to confirm the incorporation of the array of LacO repeats and by sequencing to confirm the presence of the genomic DNA sequence.

#### HAC formation assays

1 × 10^5^ HT1080^Dox-inducible mCherry-LacI-HJURP^ cells (wild-type or CENP-B knockout) were seeded into 4 wells of a 6-well plate. The next day (when cells were ∼50%–70% confluent), the media was changed, and 2 μg/mL doxycycline was added to 2 wells to induce the expression of mCherry-LacI-HJURP. Immediately after doxycycline addition, 1 μg of BAC^LacO^ DNA was transfected into each well using FuGENE 6 (Promega) at a 6:1 ratio (FuGENE 6:DNA). 24 h later, cells were split into a 15 cm plate with media lacking doxycycline, and 48 h post-transfection, 800 μg/mL G418-S was added. Cells were maintained in 800 μg/mL G418-S during clonal growth. After ∼2 weeks, clones were isolated using cloning disks made of Whatman No. 1 paper and expanded. IF-FISH was performed on each clonal cell line. At least 20 cells from each clone were assessed for the presence of a HAC, an integration event, or the absence of a detectable signal. A “HAC” designation was given if the cell contained a chromosome in which the BAC probe signal localized to > 50% of the DAPI-stainable region on the chromosome and colocalized with CENP-A signal; an “integration” designation was given if the cell contained a chromosome in which BAC probe signal localized to the DAPI-stainable region on the chromosome but did not colocalize with CENP-A signal; and a “no signal” designation was given if the cell did not contain a BAC probe signal on any DAPI-stainable region other than the endogenous region on the host chromosome. The percentage of cells containing a HAC was calculated by dividing the number of cells with a “HAC” designation by the number of cells assessed for the presence of a HAC, integration event, or the absence of a detectable signal for each clone.

Once each cell was given a “HAC,” “integration,” or “no signal” designation, the clones themselves were categorized as HAC-positive, integration-positive, or lacking a detectable signal. HAC-positive clones were those that had ≥ 20% of cells with the “HAC” designation, and integration-positive clones were those that had ≥ 20% of cells with the “integration” designation. In most of these clones, the majority of the remaining cells had a “no signal” designation; however, in the rare case that a clone had ≥ 20% of cells with the “HAC” designation and ≥ 20% of cells with the “integration designation,” the clone was categorized based on the highest majority. The percentage of clones containing a HAC, integration event, or no signal was calculated by dividing the number of clones with the specified categorization by the number of the clones screened in the experiment.

#### HAC maintenance assays

Wild-type HT1080^Dox-inducible mCherry-LacI-HJURP^ cells and three monoclonal CENP-B knockout or mCherry-LacI-HJURP knockout HT1080^Dox-inducible mCherry-LacI-HJURP^ cell lines (all harboring a chr11 α-satellite BAC^LacO^ HAC in a proportion of cells) were cultured in the absence of G418-S selection for 60 days in triplicate. IF-FISH was performed at Day 0 and Day 60, and at least 20 cells were assessed for the presence of a HAC in each cell line at both time points. A daily HAC loss rate was determined using the following equation: N_60_ = N_0_ (1-R)^60^, where R is the daily HAC loss rate and N_0_ and N_60_ are the number of metaphase chromosome spreads containing a HAC at Day 0 and Day 60, respectively (Ebersole et al., 2000, Ikeno et al., 1998).

#### IF-FISH on metaphase chromosome spreads with BAC-specific probes

IF-FISH was performed as described (Bickmore, 1999) with some modifications. HT1080 cells were treated with 50 μM STLC (Sigma-Aldrich) for 2-4 h to arrest cells during mitosis. Mitotic cells were blown off using a transfer pipette and swollen in a hypotonic buffer consisting of a 1:1:1 ratio of 75 mM KCl, 0.8% NaCitrate, and 3 mM CaCl_2_ and 1.5 mM MgCl_2_ for 15 min. 2.5 × 10^4^ cells were cytospun onto an ethanol-washed glass slide and allowed to adhere for 2 min before permeabilizing with KCM buffer for 15 min. Cells were blocked and incubated with a mouse monoclonal anti-CENP-A antibody (Enzo; ADI-KAM-CC006-E), a rabbit polyclonal anti-CENP-B antibody (Santa Cruz Biotechnology; sc-22788), and/or mouse anti-CENP-B ascites (2D-7; Earnshaw et al., 1987). Cells were washed with KCM buffer 3x for 5 min each and then incubated with Cy3 conjugated to donkey anti-mouse, Cy3 conjugated to goat anti-rabbit, Cy5 conjugated to donkey anti-mouse, and/or Cy5 conjugated to donkey anti-rabbit (Jackson ImmunoResearch; 715-165-151; 111-165-144; 715-175-151; 711-175-152, respectively). Cells were fixed with 4% formaldehyde in PBS for 10 min before being treated with 5 μg/mL RNase A for 40 min. Cells were subjected to an ethanol series to dehydrate the cells and then denatured in 70% formamide/2x SSC at 75-77°C for 2.5 min. Cells were dehydrated with an ethanol series. A biotinylated BAC^LacO^ DNA probe was generated with a Nick Translation Kit (Roche; 10976776001) according to the manufacturer’s instructions, purified with a G-50 spin column (Illustra), and ethanol-precipitated with salmon sperm DNA (for α-satellite and non-α-satellite BAC^LacO^) and Cot-1 DNA (for non-α-satellite BAC^LacO^). Precipitated BAC^LacO^ DNA was suspended in 50% formamide/10% dextran sulfate in 2x SSC and denatured at 75-77°C for 5-10 min before being placed at 37°C for at least 20 min. 100 ng α-satellite BAC^LacO^ or 300 ng non-α-satellite BAC^LacO^ DNA probe was incubated with the cells on a glass slide at 37°C overnight in a dark, humidified chamber. The next day, slides were washed 2x with 50% formamide in 2x SSC (at 45°C for α-satellite BAC^LacO^ or 37°C for non-α-satellite BAC^LacO^) and 2x with either 0.1x SSC (at 45°C for α-satellite BAC^LacO^) or 2x SSC (at 37°C for non-α-satellite BAC^LacO^). Cells were blocked with 5% milk in PBS with 0.1% Tween 20 (PBST) for 10 min. Cells were incubated with NeutrAvidin-FITC (Thermo Fisher Scientific; 31006) diluted to 25 μg/mL in 5% milk in PBST for ∼1 h at 37°C in a dark, humidified chamber. Cells were washed 3x with 4x SSC and 0.1% Tween 20 at 45°C, DAPI-stained, and mounted on a glass coverslip with Vectashield (Vector Labs). Slides were imaged on an inverted fluorescence microscope (Leica DMI6000B) equipped with a charge-coupled device camera (Hamamatsu Photonics ORCA AG) and a 100x 1.4 NA objective lens.

#### HA-LacI and EGFP-LacI lentivirus production

HA-LacI or EGFP-LacI lentivirus was produced by co-transfecting the HA-LacI or EGFP-LacI lentiviral plasmid and two packaging plasmids, pMD2.G and psPax2 (Addgene plasmids #12259 and #12260, respectively), into 293GP cells (Morgenstern and Land, 1990) and harvesting the media 48 hours later. Specifically, a 10 cm plate of 50%–80% confluent 293GP cells was transfected with 6 μg of DNA (3 μg of the HA-LacI lentiviral vector, 750 ng pMD2.G, and 2.25 μg psPax2) and 18 μL of FuGENE 6 (Promega). The culture medium was changed 6-24 h later. 48 h post-transfection, the culture medium was harvested, filtered through a 0.45 μm filter, and stored at −80°C.

#### HA-LacI lentiviral transduction and IF-FISH on metaphase chromosome spreads

HT1080^Dox-inducible mCherry-LacI-HJURP^ cells containing a 4q21 BAC^LacO^ HAC were plated in a 6 cm plate (in the presence of 800 μg/mL G418-S) and allowed to adhere to the bottom of the plate. The next day (when cells were ∼20%–30% confluent), the culture medium was replaced with fresh medium containing 200 μL of HA-LacI lentiviral supernatant and 18 μg polybrene (Specialty Media, TR-1003-G). 24 h later, the culture medium was changed to remove the lentiviral particles and polybrene. 48 h post-transduction, cells were treated with 50 μM STLC (Sigma-Aldrich) for 2-4 h to arrest cells during mitosis prior to cytospinning onto ethanol-washed glass slides to generate metaphase chromosomes spreads.

IF-FISH was carried out on metaphase chromosome spreads as described above with some modifications. A FISH probe comprised of biotinylated CENP-A ChIP DNA was generated by PCR-amplifying CENP-A ChIP DNA with GoTaq DNA polymerase (Promega, M3001), GoTaq reaction buffer, a 4:4:4:3:1 mixture of dCTP:dGTP:dATP:dTTP:biotin-11-dUTP (Thermo Scientific, R0081), and primers targeting TruSeq adapters ligated to the ends of CENP-A ChIP DNA fragments. The biotinylated CENP-A ChIP DNA was purified using a PCR purification kit (QIAGEN) and ethanol-precipitated with salmon sperm DNA. Precipitated CENP-A ChIP DNA was suspended in 50% formamide/10% dextran sulfate in 2x SSC and denatured at 75-77°C for 5-10 min before being placed at 37°C for at least 20 min. 50 ng CENP-A ChIP DNA probe was incubated with the cells on a glass slide at 37°C overnight in a dark, humidified chamber. The remaining steps of IF-FISH were carried out as described above, with wash conditions used for non-α-satellite BAC^LacO^ FISH probes. Slides were imaged on an inverted fluorescence microscope (Leica DMI6000B) equipped with a charge-coupled device camera (Hamamatsu Photonics ORCA AG) and a 100x 1.4 NA objective lens.

#### EGFP-LacI lentiviral transduction and IF-FISH on metaphase chromosome spreads with a telomere-specific probe

HT1080^Dox-inducible mCherry-LacI-HJURP^ cells containing a 4q21 BAC^LacO^ HAC were plated in a 6 cm plate (in the presence of 800 μg/mL G418-S) and allowed to adhere to the bottom. The next day (when cells were ∼20%–30% confluent), the culture medium was replaced with fresh medium containing 200 μL of EGFP-LacI lentiviral supernatant and 18 μg polybrene (Specialty Media, TR-1003-G). 24 h later, the culture medium was changed to remove the lentiviral particles and polybrene. 48 h post-transduction, cells were treated with 50 μM STLC (Sigma-Aldrich) for 2-4 h to arrest cells during mitosis prior to cytospinning onto ethanol-washed glass slides to generate metaphase chromosomes spreads.

IF-FISH was carried out on metaphase chromosome spreads as described above with some modifications. Cells were blocked for 10 min and incubated with a mouse monoclonal anti-CENP-A antibody (Enzo; ADI-KAM-CC006-E) and a rabbit polyclonal anti-GFP antibody (made in-house). Spreads were washed with KCM buffer 3x for 5 min each and then incubated with a Cy5-conjugated donkey polyclonal anti-mouse antibody (Jackson ImmunoResearch; 715-175-151) and FITC-conjugated goat polyclonal anti-rabbit antibody (Jackson ImmunoResearch; 111-095-144). Spreads were fixed with 4% formaldehyde in PBS for 10 min before being treated with 5 μg/mL RNase A for 40 min. Cells were subjected to an ethanol series (70%, 95%, 100%) to dehydrate the cells and then denatured in a hybridization mix [10 mM Tris-HCl pH 7.2, 70% formamide, 0,5% blocking reagent (Roche; 11096176001)] containing the telomere-specific FISH probe, TelC-Cy3 (PNA Bio; P1002), for 7 min at 80°C on a hot plate. The probe was incubated with cells overnight at room temperature in a dark humidified chamber, and then slides were dehydrated in an ethanol series before mounting with vectashield (Vector Labs). HACs were identified by the presence of the BAC probe signal on > 50% of the DAPI-stainable region on the chromosome. A HAC was determined to lack telomeres if there was no FISH signal on the HAC that could be distinguished from background signal.

#### Immunoblots

Whole cell lysates were collected from the indicated cell lines, separated by SDS-PAGE, transferred onto nitrocellulose membranes (BioRad), blocked with 5% milk for 1 h at room temperature, and probed with the following primary antibodies overnight at 4°C: rabbit anti-HJURP (generated against a C-terminal fragment; 1 μg/mL; Barnhart et al., 2011), human ACA (Antibodies Incorporated 15-235, 1:500), and mouse monoclonal anti-α-tubulin (Sigma-Aldrich T9026, 1:4000). The next day, blots were washed 3x in PBST and probed with the following secondary antibodies for 1-2 h at room temperature: horseradish peroxidase conjugated to donkey anti-rabbit (GE Healthcare, NA934V; 1:2,000), horseradish peroxidase conjugated to donkey anti-human (Jackson ImmunoResearch 109-035-149; 1:10,000), horseradish peroxidase conjugated to donkey anti-mouse (GE Healthcare, NA931V; 1:2,000). Blots were washed 3x in PBST and incubated with Amersham ECL detection kit (GE Healthcare). Blots were imaged using chemiluminescence with ImageQuant 400 (GE Healthcare).

#### Southern blots

Genomic DNA from HAC clones and the parental cell lines were prepared in agarose plugs and digested overnight with either FseI (NEB; R0588L) or BamHI (NEB; R0136S) at 37°C. Digested DNA was separated via CHEF electrophoresis (Bio-Rad, CHEF Mapper; autoprogram 5-500 kb range) over 16 h before being transferred to a membrane (Amersham Hybond-N+) and blot-hybridized with a 74 bp probe that binds to the LacO sequence. The LacO-specific probe was amplified via PCR using the LacO plasmid used in the Cre-Lox recombination reactions as a template and labeled with ^32^P (forward primer: 5′-AGTGGACATTTCGACCACAT-3′; reverse primer: 5′-ATTTTTATGGGCCACAAATT-3′; LacO probe: 5′-AGTGGACATTTCGACCACATTTTGTGGCCACATGTGGAATTGTGAGCGGATAACAAAATTTGTGGCCCATAAAAAT-3′).

The blot was incubated for 2 h at 65°C in hybridization buffer (0.5 M Na-phosphate with 7% SDS and 100 μg/ml of unlabeled salmon sperm carrier DNA). The labeled probe was denatured in a boiling water bath for 5 min before snap-cooling on ice. The probe was added to hybrization buffer and hybridized to the blot for 48 h at 65°C. The blot was washed with 2x SSC with 0.05% SDS for 20 min at room temperature and then washed 4x in 2x SSC with 0.05% SDS for 5 min at 60°C. Finally, the blot was exposed to X-ray film for 3 days at −80°C. Blots were imaged with a BioRad ChemiDoc MP.

#### Native CENP-A ChIP

CENP-A ChIP was performed as described (Hasson et al., 2013) with some modifications. 3-4 × 10^7^ cells were collected and resuspended in 2 mL of ice-cold buffer I (0.32 M sucrose, 15 mM Tris, pH 7.5, 15 mM NaCl, 5 mM MgCl_2_, 0.1 mM EGTA, 0.5 mM DTT, 0.1 mM PMSF, 1 mM leupeptin/pepstatin, and 1 mM aprotinin). 2 mL of ice-cold buffer I supplemented with 0.1% IGEPAL was added, and samples were placed on ice for 10 min. The resulting 4 mL of nuclei were gently layered on top of 8 mL of ice-cold buffer III (1.2 M sucrose, 60 mM KCl, 15 mM, Tris pH 7.5, 15 mM NaCl, 5 mM MgCl_2_, 0.1 mM EGTA, 0.5 mM DTT, 0.1 mM PMSF, 1 mM leupeptin/pepstatin, and 1 mM aprotinin) and centrifuged at 10,000 X *g* for 20 min at 4°C. Pelleted nuclei were resuspended in buffer A (0.34 M sucrose, 15 mM HEPES, pH 7.4, 15 mM NaCl, 60 mM KCl, 4 mM MgCl_2_, 1 mM DTT, 0.1 mM PMSF, 1 mM leupeptin/pepstatin, and 1 mM aprotinin) to 400 ng/μL. Nuclei were frozen on dry-ice and stored at −80°C. MNase (Affymetrix) digestion reactions were carried out on 300 μg chromatin, using 0.8–2.5 U/μg chromatin in buffer A supplemented with 3 mM CaCl_2_ for 10 min at 37°C. The reaction was quenched with 10 mM EGTA on ice and centrifuged at 500 X *g* for 7 min at 4°C. The chromatin was resuspended in 10 mM EDTA, pH 8.0, 1 mM PMSF, 1 mM leupeptin/pepstatin, and 1 mM aprotinin and rotated at 4°C for 2 h. The mixture was adjusted to 500 mM NaCl, allowed to rotate for another 45 min and then centrifuged at max speed (21,100 X *g*) for 5 min at 4°C, yielding nucleosomes in the supernatant. Chromatin was diluted to 100 ng/μl with buffer B (20 mM Tris, pH 8.0, 5 mM EDTA, 500 mM NaCl and 0.2% Tween 20) and precleared with 100 μL 50% protein G Sepharose bead (GE Healthcare) slurry for 20 min at 4°C, rotating. Precleared supernatant (10–20 μg bulk nucleosomes) was saved for further processing. To the remaining supernatant, 20 μg mouse monoclonal anti-CENP-A antibody [20 μg, (Abcam ab13939 or Enzo ADI-KAM-CC006-E)] was added and rotated overnight at 4°C. Immunocomplexes were recovered by addition of 200 μL 50% protein G Sepharose bead slurry followed by rotation at 4°C for 3 h. The beads were washed 3x with buffer B and once with buffer B without Tween. For the input fraction, an equal volume of input recovery buffer (0.6 M NaCl, 20 mM EDTA, 20 mM Tris, pH 7.5, and 1% SDS) and 1 μL of RNase A (10 mg/mL) was added, followed by incubation for one hour at 37°C. Proteinase K (100 μg/ml, Roche) was then added, and samples were incubated for another 3 h at 37°C. For the ChIP fraction, 300 μL of ChIP recovery buffer (20 mM Tris, pH 7.5, 20 mM EDTA, 0.5% SDS and 500 μg/mL Proteinase K) was added directly to the beads and incubated for 3–4 h at 56°C. The resulting Proteinase K–treated samples were subjected to a phenol-chloroform extraction followed by purification with a QIAGEN PCR purification column. Unamplified bulk nucleosomes or ChIP DNA was analyzed by using an Agilent Bioanalyzer instrument and a 2100 High Sensitivity Kit.

#### Next-generation sequencing and data processing

Sequencing libraries were generated and barcoded for multiplexing according to Illumina recommendations with minor modifications. Briefly, 5–10 ng input or ChIP DNA was end-repaired and A-tailed. Illumina TruSeq adaptors were ligated, libraries were size-selected to exclude polynucleosomes, and the libraries were PCR-amplified using KAPA DNA polymerase. All steps in library preparation were carried out using New England BioLabs enzymes. Resulting libraries were submitted for 75-bp, single-end Illumina sequencing on a NextSeq 500 instrument.

Single-end sequencing reads were subjected to adaptor trimming using cutadapt (Martin, 2011) and normalized to enable cross-dataset comparisons. Reads were aligned to human genome assembly hg38 and a custom reference genome (consisting of the BAC^LacO^ backbone and 256 LacO repeats, see below) in parallel using Bowtie 2 (Langmead and Salzberg, 2012). Reads aligning to chromosome 4 or the custom reference genome were extracted using SAMtools (Li et al., 2009) and converted to a bedGraph using the bamCoverage function in deepTools (Ramírez et al., 2014) with a bin size of 100 bp. The bamCoverage function generates a histogram of the number of reads for each 100-bp bin. BedGraphs were uploaded to UCSC Genome Browser to visualize read alignment data.

#### Custom reference genome

An index of the custom reference genome consisting of the BAC^LacO^ backbone and 256 LacO repeats (21,676 bp) was built from a FASTA file using Bowtie 2 (Langmead and Salzberg, 2012), and reads were aligned to the custom reference genome as described above.

To build and display the custom reference genome on the UCSC Genome Browser, a 2bit file was constructed from the FASTA file using the kentUtils source program faToTwoBit, available from the UCSC Genome Browser. An AGP file was built from the FASTA file using the kentUtils makeDb program hgFakeAgp, also available from the UCSC Genome Browser. The assembly track was constructed directly from the AGP file, generating a BED file and a bigBed file. All files (FASTA, 2bit, AGP, BED, and bigBed) were organized into folders with hub.txt, genomes.txt., groups.txt, and trackDb.txt files to generate the assembly hub, according to UCSC Genome Browser Wiki Assembly Hub’s webpage (http://genomewiki.ucsc.edu/index.php/Assembly_Hubs). The assembly hub files are publicly available at the following URL: https://eichlerlab.gs.washington.edu/help/glogsdon/public_html/customassemblies/BB1067bbLacO256x/.

#### CNV analysis

CNV analysis was performed as described previously with some modifications (Xie and Tammi, 2009). CENP-A ChIP and Input reads mapping to human genome assembly hg38 were subjected to a cnv-seq.pl script, which compares the number of reads in the ChIP and Input samples within a 5000 bp sliding window. Data were plotted in R using the ggplot2 package, where each data point represents the average fold-enrichment of a 5000 bp region with overlapping bins of ± 2500 bp (for example, bin 1 = 1-5000 bp; bin 2 = 2501-7500bp; bin 3 = 5001-10000 bp) for all annotated chromosomes.

#### Distribution of CENP-A ChIP reads within 4q21 BAC^LacO^ HACs

The distribution of CENP-A ChIP reads within the 4q21 BAC^LacO^ HACs was calculated by quantifying the mean number of CENP-A reads in the HAC-positive clone and the parental cell line within a 5000-bp window for the following regions: 4q21, vector backbone, LacO repeats, and the remaining hg38 genome. Regions in which the mean number of reads in the HAC-positive cell line was > 3 SDs above the mean number of reads in the parental cell line and had a minimum of 0.000025% of mapped reads were considered to be significantly enriched with CENP-A.

#### HAC read junction analysis

Junctions between the 4q21 sequence and α-satellite were evaluated across the CENP-A ChIP Illumina read dataset using a strategy to detect reads with exact matches to both α-satellite DNA and the HAC. We constructed a sequence database of α-satellite-containing reads using two methods: 1) Reads were mapped (BWA-MEM, standard parameters; Li, 2013, Li and Durbin, 2010) to the GRCh38 human assembly (including alternative assemblies), which contains human α-satellite sequence models in each centromeric region (Miga et al., 2014; BioProject: PRJNA193213). Reads were identified as containing α-satellite if they overlapped with sites (BEDTools: intersect; Quinlan and Hall, 2010) in the genome previously annotated as α-satellite. The UCSC table browser (Karolchik et al., 2004) was used to obtain a bed file of all sites annotated as ALR/Alpha Satellite. 2) In addition to our mapping strategy, we characterized α-satellite using a previously published WGS read database of α-satellite, representing 2.6% of sequences from the HuRef genome (Hayden et al., 2013, Levy et al., 2007). To do so, we identified a listing of ∼8 million α-satellite-specific 18-mers (i.e., 18-mers that did not contain an exact match with any sequence in the HuRef genome, or GRCh38 reference assembly, outside of sequences of known α-satellite). Illumina reads were defined as containing α-satellite if they contained an exact match with at least five 18-mers specific to α-satellite, as determined empirically. Comparisons between the mapping and k-mer-based strategies were highly concordant. To confirm the presence of a junction between the 4q21 sequence and α-satellite, we reformatted our α-satellite sequence database into all possible 36-mers in both orientations (Marçais and Kingsford, 2011) and identified exact matches with 36-mers specific to 4q21 (GRCh38; chr4:86937133-87119178) within the same read.

#### Deep-sequencing and chimeric read analysis

Illumina-prepped libraries for clone 11 and the CENP-B knockout parental cell line were deep-sequenced on the NextSeq 500 to generate 150-bp single-end reads, thereby increasing the read depth for these samples by ∼40X. Reads were processed to trim adapters via cutadapt (Martin, 2011) and aligned to hg38 using BWA-MEM (Li and Durbin, 2010). Chimeric reads from the regions flanking the CENP-A-enriched region in 3q13 or 9q22 (+/− 5 or 15 kb for 3q13 and 9q22, respectively) were extracted using the “SA” flag and mapped to alternate locations using BLASTn (Altschul et al., 1990).

#### IF on chromatin fibers

HT1080^Dox-inducible mCherry-LacI-HJURP^ cells containing a 4q21 BAC^LacO^ HAC were plated in a single well of a 6-well plate (in the presence of 800 μg/mL G418-S) and allowed to adhere to the bottom of the plate. The next day (when cells were ∼20%–30% confluent), the culture medium was replaced with fresh medium containing 500 μL of HA-LacI lentiviral supernatant and 8 μg polybrene (Specialty Media, TR-1003-G). 24 h later, the culture medium was changed to remove the lentiviral particles and polybrene. 48 h post-transduction, chromatin fibers were prepared as described with modifications (Iwata-Otsubo et al., 2017, Sullivan, 2010). Briefly, cells were collected and swollen in a hypotonic buffer consisting of a 1:1:1 ratio of 75 mM KCl, 0.8% NaCitrate, and dH_2_O for 5 min. 3.5 × 10^4^ cells were cytospun onto an ethanol-washed glass slide at 800 rpm for 4 min with high acceleration and allowed to adhere for 1 min before immersing in a salt-detergent-urea lysis buffer (25 mM Tris pH 7.5, 0.5 M NaCl, 1% Triton X-100, and 0.3 M urea) for 15 min at room temperature. The slide was slowly removed from the lysis buffer over a time period of 33 s and subsequently washed in PBS, incubated in 4% formaldehyde in PBS for 10 min, and washed with PBS and 0.1% Triton X-100. The slide was rinsed in PBS and 0.05% Tween-20 for 3 min blocked for 30 min with IF block (2% FBS, 2% BSA, 0.1% Tween-20, and 0.02% NaN_2_), and then incubated with a mouse monoclonal anti-CENP-A antibody (1:200 Enzo; ADI-KAM-CC006-E) and rabbit monoclonal anti-HA antibody (1:200, Cell Signaling Technology, 3724S) for 3 h at room temperature. Cells were washed 3x for 5 min each in PBST and then incubated with Cy3 conjugated to donkey anti-mouse (1:200) and FITC goat anti-rabbit (1:200) for 1.5 h. Cells were washed 3x for 5 min each in PBST, fixed for 10 min in 4% formaldehyde, and washed 3x for 1 min each in dH_2_O before mounting in vectashield containing 5 μg/ml DAPI. Slides were imaged on an inverted fluorescence microscope (Leica DMI6000B) equipped with a charge-coupled device camera (Hamamatsu Photonics ORCA AG) and a 100x 1.4 NA objective lens.

### Quantification and Statistical Analysis

#### Quantification of CENP-A, CENP-B, and CENP-A ChIP FISH probe intensity

All images were cropped to contain a single metaphase chromosome spread (i.e., a spread of metaphase chromosomes coming from a single cell) using ImageJ (version 1.46r; Schneider et al., 2012).

The fluorescence intensity of CENP-A at the HAC was measured by placing a 7 X 7 pixel box around each CENP-A signal and measuring the total pixel intensity within the box. The mean CENP-A fluorescence intensity at centromeres was measured using an ImageJ macro, CRaQ_v1.12chromosomespreads, a modified version of the ImageJ macro, CRaQ (Bodor et al., 2012), in which a 7 X 7 pixel box was placed around the centroid position of each CENP-A signal and the total pixel intensity within the box was measured and averaged over the total number of centromeres in each cell. The ratio of the average CENP-A intensity at the HAC to the average CENP-A intensity at endogenous centromeres was calculated for at least 20 chromosome spreads and presented in a plot using Prism 6.

The fluorescence intensity of CENP-B at the HAC was measured by placing a 20 X 20 pixel box around the CENP-B signal and measuring the total pixel intensity within the box. The mean CENP-B fluorescence intensity at centromeres was measured using an ImageJ macro, CRaQ_v1.12CBonchromosomespreads, a modified version of the ImageJ macro, CRaQ (Bodor et al., 2012), in which a 20 X 20-pixel box was placed around the centroid position of the CENP-B signal and the total pixel intensity within the box was measured and averaged over the total number of centromeres in each cell. The ratio of the average CENP-B intensity at the HAC to the average CENP-B intensity at endogenous centromeres was calculated for at least 20 chromosome spreads and presented in a plot using GraphPad Prism.

The fluorescence intensity of the CENP-A ChIP FISH probe at the HAC was measured by placing a 22 X 22 pixel box around the CENP-A ChIP FISH signal and measuring the total pixel intensity within the box. The mean CENP-A ChIP FISH probe intensity at centromeres was measured using an ImageJ macro, CrAQ_v1.12CBonchromosomespreads, in which a 22 X 22 pixel box was placed around the centroid position of the CENP-A ChIP FISH signal (identified with a threshold factor of 1.1). The total pixel intensity within the box was measured and averaged over the total number of centromeres in each cell. The ratio of the average CENP-A ChIP FISH probe intensity at the HAC to the average intensity at endogenous centromeres was calculated for at least 15 chromosome spreads and presented in a plot using GraphPad Prism.

Both CRaQ_v1.12chromosomespreads and CRaQ_v1.12CBonchromosomespreads ImageJ macros are available upon request.

#### Quantification of HAC chromatin fibers

HAC fibers were identified by the colocalization of HA signal with DAPI. Quantification of the fibers was performed if the fiber was sufficiently stretched (i.e., the HA signal was interrupted by regions lacking signal, and CENP-A was observed between HA signals on the DAPI-stained fiber). The number of regions containing CENP-A signal between HA signals was divided by the total number of regions between HA signals to determine the fraction of 4q21 BAC^LacO^ copies within the HAC occupied by CENP-A. Since our experimental approach must preserve chromatin while stretching chromosomes, many HACs in our analysis have regions where LacO arrays from neighboring copies of 4q21 BAC^LacO^ cannot be resolved from one another. Thus, our calculation for the fraction of 4q21 BAC^LacO^ copies occupied by CENP-A is likely an overestimate of the actual fraction.

#### Statistical information

The statistical significance of the difference between the mean for HAC maintenance assay and CENP-A intensity datasets was measured using unpaired, two-tailed t tests, and the statistical significance of the difference of the mean between CENP-B intensity and CENP-A ChIP FISH probe intensity datasets were measured using one-sample t tests with a hypothetical mean of 0. The p values resulting from these t tests are stated in the relevant figure legend or text. If the p value was < 0.05, it was marked as ‘significant’; however, if the p value ≥ 0.05, it was marked as ‘not significant’ (n.s.).

### Data and Code Availability

Next-generation sequencing data for 4q21 HAC clones 1, 4, 7, and 11-17 are available at BioProject: PRJNA487691. All custom code, including those used to determine the copy number and distribution of CENP-A ChIP reads within the 4q21 BAC^LacO^ HACs, are available from the authors upon request.
