# Supplementary material for: Human Artificial Chromosomes that Bypass Centromeric DNA
Source: Cell. 2019 Jul 25;178(3):624–639.e19. doi: 10.1016/j.cell.2019.06.006 (PMC6657561; doi:10.1016/j.cell.2019.06.006)
Supplement: Document S1. Tables S1–S6 [file mmc1.pdf]

Cell, Volume *178*

Supplemental Information

Human Artificial Chromosomes

that Bypass Centromeric DNA

Glennis A. Logsdon, Craig W. Gambogi, Mikhail A. Liskovykh, Evelyne J. Barrey, Vladimir Larionov, Karen H. Miga, Patrick Heun, and Ben E. Black

Logsdon et al., Table S1

BAC	Neocentromere to which the BAC insert is proximal	Coordinates of BAC insert in hg38	Coordinates of neocentromere in hg38	Distance between BAC insert and neocentromere
4q21 BAC	PD-NC4	chr4:86,937,133-87,119,178 [~182 kb]	chr4:87,228,848 -87,628,848	109,670 bp
4q22 BAC	PD-NC4	chr4:87,260,058-87,426,318 [~166 kb]	chr4:87,228,848 -87,628,848	Located within
4q28 BAC	---	chr4:128,716,484-128,873,622 [~157 kb]	---	---
8q21 BAC	MS4221	chr8:85,793,719-85,805,252 [~183 kb]*	chr8:85,587,771 -85,887,771	Located within

Table S1. Genomic Sequence Locations of the Non-α-satellite BAC^{LacO} Constructs, Related to Figure 3.
Chromosomal coordinates of the genomic sequences in the non-α-satellite BAC^{LacO} constructs relative to neocentromeres (Hasson et al., 2013). The asterisk indicates a construct (8q21 BAC^{LacO}) that consists almost entirely of ~12 kb repeats. In the hg38 reference genome, the 8q21 BAC^{LacO} insert aligns to a single repeat; however, the actual size of the 8q21 BAC^{LacO} genomic insert is ~183 kb.

Logsdon et al., Table S2

Clone	Distribution of CENP-A ChIP reads			
	4q21	Backbone	LacO	Genomic
1	81.7%	17.6%	0.3%	0.4%
11	89.1%	5.1%	0.6%	5.2%
12	68.2%	31.0%	0.7%	0.1%
14	91.4%	5.6%	1.1%	1.9%
4	4.9%	0.7%	0.1%	94.3%
7	67.0%	6.2%	0.2%	26.6%
13	51.2%	6.5%	0.5%	41.8%
17	32.8%	4.7%	0.5%	62.0%

Experiment
WT, +LacO
WT, –LacO
CENP-B KO, +LacO

Table S2. The Distribution of CENP-A ChIP Reads in the CENP-B- and Functional α-Satellite-Negative 4q21 HACs, Related to Figure 4.
Table summarizing the distribution of CENP-A ChIP reads in each of the 4q21 HACs lacking CENP-B and functional α-satellite sequences. The top 4 clones have a centromere positioned almost entirely on the 4q21 and vector backbone sequences, whereas the bottom four clones have a centromere that is positioned on the 4q21 sequence, vector backbone, and host chromosome sequences (as shown in the “Genomic” column).

Logsdon et al., Table S3

Clone	Average copy number of the 4q21 sequence within the HAC	Average # of HACs / cell	Average size of HAC (Mb)
1	53	1.2	9.0
11	55	2.1	5.3
12	41	1.2	6.9
14	46	1.4	6.7

Experiment
WT, +LacO
CENP-B KO, +LacO

Table S3. Calculation to Determine the Average Size of Each HAC Whose Centromere is Essentially Comprised of 4q21 BAC^{LacO}, Related to Figure 5. Table summarizing the average size of the 4q21 BAC^{LacO} HACs formed without acquiring CENP-B- or CENP-A-associated sequences. Because these clones are comprised almost entirely of 4q21 BAC^{LacO} sequences, their size can be accurately calculated using the following formula: (CN_H x 0.203 Mb) / H, where CN_H is the average copy number of the 4q21 sequence within the HAC, and H is the average # of HACs/cell. CN_H, itself, is calculated using the following formula: FE x CN_P, where FE is the average fold-enrichment of the 4q21 sequence in the HAC clone (shown in Fig. S5A) and CN_P is the average raw copy number of the parental cell line (2.7, as determined by IF-FISH). All of the 4q21 BAC^{LacO} HACs lacking acquired genomic sequences range in size from 5.3-9.0 Mb.

Logsdon et al., Table S4

Clone	Location of genomic CENP-A enrichment	
	α-satellite	non-α-satellite
4	chr10:41,544,942-42,110,942	chr6:168,294,667-168,314,200<br>chr16:87,197,849-87,199,200
7	chr16:34,245,822-34,289,488	chr5:47,152,890-47,303,000
13	chrX:62,499,084-62,499,160	--
17	chr10:41,487,500-41,767,500	chr3:116,741,534-116,745,800<br>chr9:99,135,406-99,156,500

Experiment
WT, +LacO
WT, –LacO
CENP-B KO, +LacO

Table S4. Genomic Coordinates of the CENP-A-Enriched Regions in the 4q21 HAC Clones that Have Acquired Endogenous Sequences from Host Chromosomes, Related to Figure 5.
Table summarizing the genomic location of CENP-A enrichment in 4q21 HACs lacking CENP-B and functional α-satellite sequences. Clone 13 has acquired only α-satellite sequences, while clones 4, 7, and 17 have acquired both α-satellite and non-α-satellite sequences during HAC formation.

Logsdon et al., Table S5

Locus	Size (bp)	% GC	% SINE	% LINE	% LTR	% DNA repeat	% Simple repeat	% Low complexity repeat	% Satellite
4q21	182,046	41.1%	20.5%	9.5%	1.8%	4.0%	0.6%	0.5%	0.0%
4q21 upstream	182,046	40.5%	21.9%	16.9%	9.0%	1.7%	0.6%	0.7%	0.0%
4q21 downstream	182,046	39.1%	19.2%	15.6%	10.4%	5.1%	0.7%	0.8%	0.0%
3q13	4,267	39.2%	0.0%	35.2%	2.1%	0.0%	1.1%	0.8%	0.0%
3q13 upstream	4,267	34.0%	8.2%	17.0%	0.0%	5.7%	1.1%	0.0%	0.0%
3q13 downstream	4,267	39.6%	0.0%	83.2%	0.0%	0.0%	0.6%	0.0%	0.0%
9q22	21,095	37.4%	10.3%	17.67%	2.8%	2.9%	0.3%	0.4%	0.0%
9q22 upstream	21,095	37.4%	10.7%	13.7%	2.9%	5.2%	0.4%	0.5%	0.0%
9q22 downstream	21,095	43.1%	14.1%	31.2%	8.7%	3.0%	1.4%	0.1%	0.0%
4q22	166,262	40.6%	21.4%	13.3%	21.9%	4.0%	0.7%	0.6%	0.0%
4q22 upstream	166,262	39.0%	18.7%	15.4%	4.3%	4.2%	0.7%	0.6%	0.0%
4q22 downstream	166,262	39.0%	17.6%	20.7%	8.8%	5.3%	0.9%	0.8%	0.0%
4q28	157,140	43.0%	25.3%	8.1%	8.1%	2.2%	0.7%	0.4%	0.0%
4q28 upstream	157,140	41.8%	21.2%	12.5%	19.1%	2.6%	0.6%	0.5%	0.0%
4q28 downstream	157,140	35.7%	8.9%	40.6%	3.6%	2.7%	0.4%	0.5%	0.0%
8q21	11,535	47.5%	10.2%	38.3%	0.8%	1.9%	0.3%	0.2%	0.0%
8q21 upstream	11,535	47.0%	10.2%	39.5%	0.8%	7.1%	0.3%	0.2%	0.0%
8q21 downstream	11,535	47.1%	9.5%	38.3%	0.8%	6.2%	0.3%	0.0%	0.0%
Whole genome	3,099,922,541	40.9%	12.6%	19.9%	8.5%	3.2%	0.8%	0.5%	2.1%

Table S5. Genomic Content of the Sequences Tested in this Study and Those Acquired During HAC formation, Related to Figure 5.
The GC content and abundance of repeat elements is indicated for the non-α-satellite sequences tested in this study and their flanking regions, as well as sequences acquired by clone 17 during HAC formation. A whole-genome analysis is also shown for comparison. We did not observe a clear enrichment for particular repeat elements within the 4q21, 3q13, and 9q22 sequences compared to their flanking regions, other sequences tested in this study, or the whole genome, so we conclude that there is no clear correlation between repeat element abundance and the formation of centromeric chromatin. If there is a genetic code that positively affects the assembly of centromeric chromatin on HAC templates comprised of non-centromeric DNA repeats, it is too cryptic to be ascertained from standard analysis of our data. However, one can imagine many genetic elements absent in our tested sequences (e.g. strong promoters, super enhancers, etc.) that could have negatively affected centromeric chromatin assembly.

Logsdon et al., Table S6

Clone	Percentage of cells containing a HAC	Figure referenced
1	85%	3B,C; 4B-F; 5A,D; 6; S3; S4A; S5A,C; S6B,C
2	100%	3B; 4B,F; S3A
3	95%	3B,C; 4B,D,F; 5D,E; S3; S6D
4	100%	3B; 4B,D,F; S3A,B; S4B; S5B
5	80%	3B; 4B,F; S3A
6	100%	3B; 4B,F
7	100%	3D; 4B,F; S3A; S4B; S5B
8	55%	3D; 4B,C,F
9	90%	3D; 4B,F
10	55%	3D,E; 4B,F; S3A
11	75%	3F; 4D,F; 5A,D; S3B-D; S4A; S5A,C; S6B
12	100%	3F; 4D,F; 5A,D; S3B-D; S4A; S5A,C; S6B
13	90%	3F,G; 4D,F; 5B; S3B-D; S4B; S5B
14	75%	3F; 4D,F; 5A,D; S3B-D; S4A; S5A,C; S6B
15	95%	3F; 4D,F
16	85%	3F,G; 4D-F
17	100%	3F; 4D,F; 5B; S3B-D; S4B; S5B,C; S6A
18	95%	1D,F
19	95%	1D-F
20	100%	1D,F
21	65%	1D,F
22	85%	1D,F
23	85%	1D,F
24	95%	1D,F-H; 2F,G,I,J
25	55%	1D,F
26	60%	1D,F
27	100%	1D-F; 2G
28	55%	1D,F
29	100%	1G,H
30	90%	1G,H
31	95%	1G,H
32	95%	2D,F,G
33	85%	2D,F
34	100%	2D,F,G
35	95%	2D-G
36	85%	2D,F
37	95%	2D,F
38	85%	2D,F
39	55%	2D,F
40	65%	2D,F
41	100%	2I,J; S1
42	90%	2I,J
43	90%	2I,J

Experiment
4q21, WT, +LacO
4q21, WT, –LacO
4q21, CENP-B KO, +LacO
chr7 α-sat, WT, +LacO
chr11 α-sat, WT, +LacO
chr11 α-sat, CENP-B KO, +LacO

Table S6. Summary of the HAC Clones Generated in This Study, Related to STAR Methods.
All clones generated in this study have a HAC in at least 55% of cells, with the vast majority having a HAC in at least 85% of cells. The relevant figures that the HAC clones are described in is listed.
